# Voltage Imaging in *Drosophila* Using a Hybrid Chemical-Genetic Rhodamine Voltage Reporter

**DOI:** 10.3389/fnins.2021.754027

**Published:** 2021-11-16

**Authors:** Molly J. Kirk, Brittany R. Benlian, Yifu Han, Arya Gold, Ashvin Ravi, Parker E. Deal, Rosana S. Molina, Mikhail Drobizhev, Dion Dickman, Kristin Scott, Evan W. Miller

**Affiliations:** ^1^Department of Molecular and Cell Biology, University of California, Berkeley, Berkeley, CA, United States; ^2^Department of Neurobiology, University of Southern California, Los Angeles, CA, United States; ^3^Department of Chemistry, University of California, Berkeley, Berkeley, CA, United States; ^4^Department of Microbiology and Cell Biology, Montana State University, Bozeman, MT, United States; ^5^Helen Wills Neuroscience Institute, University of California, Berkeley, Berkeley, CA, United States

**Keywords:** imaging, fluorescence, voltage, *Drosophila*, neuromuscular junction (NMJ)

## Abstract

We combine a chemically-synthesized, voltage-sensitive fluorophore with a genetically encoded, self-labeling enzyme to enable voltage imaging in *Drosophila melanogaster*. Previously, we showed that a rhodamine voltage reporter (RhoVR) combined with the HaloTag self-labeling enzyme could be used to monitor membrane potential changes from mammalian neurons in culture and brain slice. Here, we apply this hybrid RhoVR-Halo approach *in vivo* to achieve selective neuron labeling in intact fly brains. We generate a *Drosophila* UAS-HaloTag reporter line in which the HaloTag enzyme is expressed on the surface of cells. We validate the voltage sensitivity of this new construct in cell culture before driving expression of HaloTag in specific brain neurons in flies. We show that selective labeling of synapses, cells, and brain regions can be achieved with RhoVR-Halo in either larval neuromuscular junction (NMJ) or in whole adult brains. Finally, we validate the voltage sensitivity of RhoVR-Halo in fly tissue *via* dual-electrode/imaging at the NMJ, show the efficacy of this approach for measuring synaptic excitatory post-synaptic potentials (EPSPs) in muscle cells, and perform voltage imaging of carbachol-evoked depolarization and osmolarity-evoked hyperpolarization in projection neurons and in interoceptive subesophageal zone neurons in fly brain explants following *in vivo* labeling. We envision the turn-on response to depolarizations, fast response kinetics, and two-photon compatibility of chemical indicators, coupled with the cellular and synaptic specificity of genetically-encoded enzymes, will make RhoVR-Halo a powerful complement to neurobiological imaging in *Drosophila*.

## Highlights

- Voltage imaging is a powerful method for interrogating neurobiology.
- Chemical indicators possess fast response kinetics, turn-on responses to membrane depolarization, and can be compatible with two-photon excitation.
- However, selective cell labeling in intact tissues and *in vivo* remains a challenge for completely synthetic fluorophores.
- Here, we show that a chemical—genetic hybrid approach in *Drosophila* enables cell-specific staining *in vivo* and voltage imaging in whole-brain explants and at neuromuscular junction synapses.

## Introduction

Voltage imaging in intact brains offers the tantalizing promise to watch, in real time, the electrical changes that underlie physiology. Approaches for voltage imaging rely on fluorescent indicators, either chemically synthesized, genetically encoded, or combinations of the two. Chemically-synthesized indicators have a storied past (Davila et al., 1973), enabling visualization of membrane potential changes in a diverse range of species and preparations (Grinvald et al., 1977, 1981; Cacciatore et al., 1999; Grinvald and Hildesheim, 2004). One serious drawback of chemically-synthesized indicators is their poor innate ability to localize to specific neurons. On the other hand, genetically-encoded voltage indicators (GEVIs) circumvent problems of localization to specific neurons. Fresh rounds of innovation improved upon first-generation GEVIs (Storace et al., 2016; Kannan et al., 2019), enhancing membrane trafficking and response kinetics, and introducing alternative approaches to fluorescent protein/voltage-sensing domain fusions, including voltage-sensitive opsins and electrochromic-FRET pairs (Lin and Schnitzer, 2016; Yang and St-Pierre, 2016).

Our group has focused on the development of chemically-synthesized voltage-sensitive fluorophores that respond to changes in membrane potential *via* a photoinduced electron transfer (PeT) based mechanism. At hyperpolarizing potentials, the voltage across the membrane accelerates PeT from one side of the molecule to the other, short-circuiting and quenching fluorescence (Liu and Miller, 2020). At depolarized potentials, PeT is slowed, and the quantum yield of the dye increases. This configuration allows fast (Beier et al., 2019), linear, turn-on responses to depolarizations (with corresponding fluorescence decreases for hyperpolarization), good signal to noise, and compatibility with 2P excitation (Kulkarni et al., 2018; Kazemipour et al., 2019). However, attempts to deploy voltage-sensitive fluorophores in brain tissues resulted in comprehensive staining of all neuronal membranes, making it difficult to visualize clear boundaries between cells or regions of the brain (Woodford et al., 2015; Kulkarni et al., 2018). Therefore, there is strong interest in developing hybrid systems in which voltage-sensitive dyes are directed to cells of interest, either *via* expression of exogenous enzymes (Ng and Fromherz, 2011; Liu et al., 2017; Sundukova et al., 2019) or *via* targeting of native ligands (Fiala et al., 2020). Other strategies involve targeting synthetic fluorophores to genetically-encoded voltage-sensitive proteins, whether opsins (Abdelfattah et al., 2019; Liu et al., 2021) or voltage-sensing domains (Deo et al., 2021).

**FIGURE 1:**
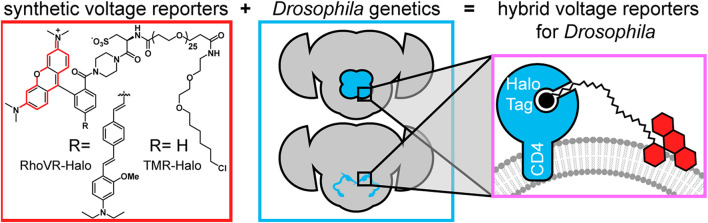
Chemical-genetic hybrids for voltage imaging in *Drosophila*. (red box) Chemically synthesized rhodamines with chloroalkane ligands will form covalent adducts with HaloTag enzymes. When the functional group R is the indicated molecular wire, the resulting RhoVR-Halo is voltage-sensitive. When R is H, the tetramethyl rhodamine-Halo is not voltage-sensitive (TMR-Halo) (teal box). The use of GAL4-UAS fly lines enables selective expression of HaloTag enzymes in defined populations of neurons. (magenta box) When HaloTag is fused with CD4, expression on the cell surface of defined neurons allows *in vivo* labeling (with either TMR-Halo or RhoVR-Halo) followed by *ex vivo* voltage imaging (with RhoVR-Halo).

We recently reported a chemical-genetic hybrid, in which a chemically-synthesized rhodamine-based voltage reporter (RhoVR) (Deal et al., 2016) attached to a flexible polyethyleneglycol (PEG) linker terminating with a chloroalkane forms a covalent bond with a cell-expressed HaloTag (Figure 1), enabling voltage imaging from defined neurons, in mouse cortical brain slices (Deal et al., 2020). This approach, RhoVR-Halo, takes advantage of the fast kinetics, linear turn-on response, and 2P compatibility of RhoVR-type indicators (Kulkarni et al., 2018; Kazemipour et al., 2019), and pairs it with the ability to target specific cells using traditional genetic methods. Other dyes, like tetramethylrhodamine (TMR)-Halo (Figure 1), can be used to provide a convenient, non-voltage sensitive stain for control experiments in the same genetic background.

The wealth of genetic tools, small brain size for optical imaging, and short generation time make *Drosophila melanogaster* an attractive model organism (Simpson, 2009; Caygill and Brand, 2016; Simpson and Looger, 2018). Genetically encoded indicators have been previously deployed in *Drosophila* and fall into two broad classes: (1) fluorescent protein (FP) fusions with voltage-sensing domains and (2) electrochromic FRET indicators (eFRET) that couple voltage-dependent changes in opsin absorbance with FRET to a fluorescent protein. FP-VSD fusions like ArcLight (Jin et al., 2012; Jourjine et al., 2016; Chen et al., 2017) or ASAP (Yang et al., 2016; Chamberland et al., 2017), have been used in multiple *Drosophila* contexts and show negative-going responses to membrane depolarizations, use “GFP”-like excitation and emission, and display non-linear response kinetics across the entire physiological range. Electrochromic-FRET indicators (Zou et al., 2014) like Ace2N-mNeon (Gong et al., 2015) or Varnam (Kannan et al., 2018) (and their chemigenetic relative, Voltron, which replaces the FP with a HaloTag) (Abdelfattah et al., 2019) have also been used in *Drosophila* and provide fast, negative-going responses to depolarizations. These types of indicators are not compatible with 2P excitation, likely owing to the complex photocycle involved in opsin-based voltage sensitivity (Maclaurin et al., 2013).

Therefore, to expand the RhoVR-Halo methodology beyond vertebrate systems, we developed a stable transgenic UAS reporter line in *Drosophila* to express HaloTag on the extracellular surface of neurons, enabling the selective staining of defined neuronal populations when crossed with existing GAL4 driver lines. When paired with voltage-sensitive RhoVR-Halo (Deal et al., 2020), HaloTag-expressing flies allow cell type-specific labeling *in vivo*, and voltage imaging in a variety of contexts, including synaptic imaging at the neuromuscular junction (NMJ) and across defined neuronal populations in fly brain explants.

## Results

### Generation of HaloTag Constructs for Expression in Flies

Although HaloTag and other self-labeling enzymes have been successfully expressed in transgenic flies, the reported lines localize HaloTag intracelluarly (Kohl et al., 2014; Sutcliffe et al., 2017; Meissner et al., 2018; Abdelfattah et al., 2019). Our first task was to generate a HaloTag that expressed on the extracellular face of membranes. Previous chemical-genetic hybrids deployed in mammalian cells used a transmembrane domain from the platelet-derived growth factor receptor (PDGFR) to localize HaloTag to the cell surface and a secretion signal peptide from immunoglobulin K (IgK) to enhance export of the construct (Deal et al., 2020). To adapt HaloTag-directed chemical-genetic hybrids for voltage imaging in *Drosophila*, we selected CD4 as a transmembrane anchor, on account of its good membrane association in *Drosophila* neurons (Han et al., 2011), fusing it to the C-terminus of the HaloTag. We sub-cloned this construct into different vectors for expression in mammalian (pcDNA3.1) and insect cells (pJFRC7) (Pfeiffer et al., 2010).

**FIGURE 2:**
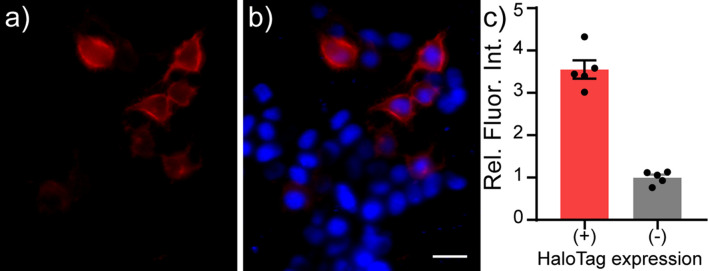
Live-cell staining of RhoVR-Halo in HEK293T cells expressing HaloTag-CD4. Epifluorescence images of HEK293T cells expressing HaloTag-CD4 (CMV promoter) and stained with RhoVR-Halo (100 nM, red) and Hoechst 33342 (1 μM, blue). Images show **(a)** RhoVR-Halo fluorescence and **(b)** an overlay of RhoVR-Halo and Hoechst fluorescence. Scale bar is 20 μm. **(c)** Plot of relative fluorescence intensity in cells expressing HaloTag vs. cells that do not express HaloTag. HaloTag (+) cells were assigned based on a threshold obtained from a non-transfected control. Data are mean ± SEM for *n* = 5 different coverslips of cells. Data points represent average fluorescence intensities of 30–40 cells.

HaloTag-CD4 shows good expression on cell surfaces. In mammalian cells, CD4 localizes to the cell surface by anti-CD4 immunocytochemistry (Supplementary Figure 1). Inclusion of the self-labeling enzyme, HaloTag, affords the opportunity to confirm not only localization, but activity of the expressed enzyme by delivering HaloTag substrates. HEK cells expressing HaloTag-CD4 and treated with RhoVR-Halo (100 nM) show good membrane localization (Figure 2a), while cells that do not express HaloTag-CD4 show approximately 3.5-fold lower fluorescence levels (Figures 2b,c). RhoVR-Halo survives fixation: following live-cell imaging, cells can be fixed and retain their RhoVR-Halo staining, which serves as a useful counterstain to the anti-CD4 immunocytochemistry (Supplementary Figure 1). Live cells labeled with RhoVR-Halo and subsequently fixed, permeabilized with detergent and assayed for CD4 *via* immunochemistry reveal the majority of CD4 is found intracellularly. Confocal imaging shows that RhoVR-Halo localizes to cell membranes (Supplementary Figures 2A–C). HaloTag-mediated labeling works with other dyes, too: HEK cells expressing HaloTag-CD4 and labeled with TMR-Halo show approximately 15-fold greater fluorescence than cells that do not express HaloTag-CD4 (Supplementary Figure 2).

**FIGURE 3:**
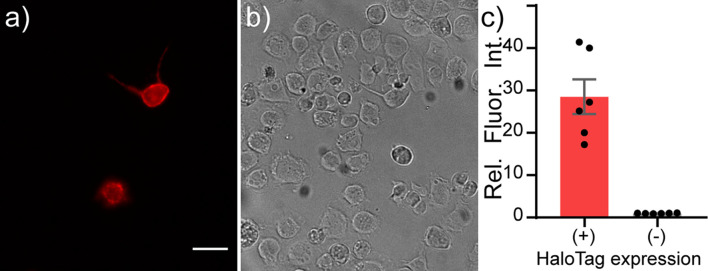
Live-cell staining of *Drosophila* S2 cells with TMR-Halo. Live-cell staining of TMR-Halo in *Drosophila* S2 cells expressing HaloTag-CD4. Epifluorescence images of *Drosophila* S2 cells transfected with tubP-GAL4 and HaloTag-CD4 UAS and **(a)** stained with TMR-Halo (100 nM). **(b)** Transmitted light image of cells in panel **(a)**. Scale bar is 20 μm. **(c)** Plot of relative fluorescence intensity in cells expressing HaloTag vs. cells that do not express HaloTag from the same cultures. HaloTag-(+) cells were assigned based on a threshold obtained from a non-transfected control. Data are mean ± SEM for *n* = 6 different coverslips.

In S2 cells, an immortalized *Drosophila* cell line, we also observe cell surface localization of HaloTag-CD4, as visualized by anti-CD4 immunocytochemistry (Supplementary Figure 3). S2 cells show similar HaloTag-CD4 dependent staining with TMR-Halo (100 nM, Figure 3 and Supplementary Figures 3A,B) with a 20-fold enhancement in fluorescence intensity in HaloTag-CD4 expressing cells compared to non-expressing cells (Figure 3c). TMR-Halo staining in S2 cells is also retained post-fixation (Supplementary Figure 3).

**FIGURE 4:**
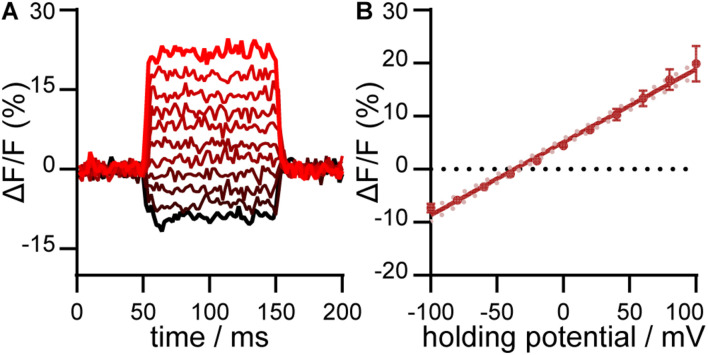
Voltage sensitivity of RhoVR-Halo in HEK293T cells expressing HaloTag-CD4. **(A)** Plot of ΔF/F vs. time for a single HEK293T cell expressing HaloTag-CD4 and stained with RhoVR-Halo. The HEK293T cell was held at –60 mV and then stepped through hyperpolarizing and depolarizing potentials, in 20 mV increments, from –100 mV to +100 mV. **(B)** Plot of ΔF/F vs. potential in mV. Data are mean ± standard error of the mean for *n* = 7 separate cells. Solid line is the line of best fit, and pink dots are 95% confidence interval.

We evaluated the voltage sensitivity of RhoVR-Halo in HaloTag-CD4 expressing HEK293T cells (Figure 4). After loading cells with RhoVR-Halo (500 nM), cells were subjected to whole-cell, patch-clamp electrophysiology. The voltage sensitivity of RhoVR-Halo in HaloTag-CD4 expressing HEK293T cells is approximately 14% per 100 mV (± 2%, SEM *n* = 7 cells). This is approximately 70% of the value we obtained when HaloTag is targeted with previously developed (Deal et al., 2020) HaloTag-pDisplay (Supplementary Figure 4).

### Validation of HaloTag-Expressing Flies

**FIGURE 5:**
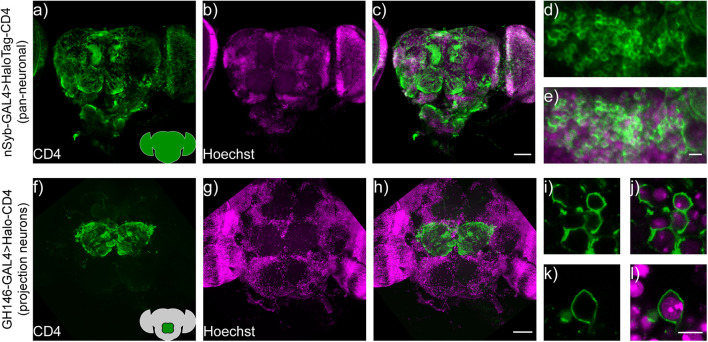
HaloTag-CD4 expression in transgenic *Drosophila*. **(a)** nSyb-GAL4, HaloTag-CD4 brains express CD4 pan-neuronally. Maximum z-projection of a confocal fluorescence microscopy stack of brain explant from either **(a–e)** nSyb-GAL4 > HaloTag-CD4 or **(f–l)** GH146-GAL4 > HaloTag-CD4, fixed and stained for an extracellular epitope of the CD4 protein (OKT4, green) and counterstained for nuclei with Hoechst 33342 (16 μm or 10 μg/mL, magenta). Scale bar is 50 μm for whole-brain images **(a–c,f–h)** and 5 μm for zoomed-in regions **(d,e,i–l)**. Insets on panels **(a,f)** show schematized brains with an approximate location of the staining for reference.

To evaluate the performance of cell surface-expressed HaloTag-CD4 in intact flies, we generated transgenic flies (BestGene Inc.) and crossed the resulting UAS-HaloTag-CD4 line with a pan-neuronal driver line, neuronal synaptobrevin-GAL4 (nSyb-GAL4) (DiAntonio et al., 1993), which was used to drive HaloTag-CD4 expression in all neurons, (Figure 5a). Brains of nSyb-GAL4 > HaloTag-CD4 flies show strong CD4 expression (Figures 5a–c). The pattern of anti-CD4 fluorescence indicates good localization to the plasma membrane (Figures 5d,e).

To evaluate labeling specificity, we expressed UAS-HaloTag-CD4 in a subset of neurons. We crossed UAS-HaloTag-CD4 flies with GH146-GAL4 flies (Stocker et al., 1997; Wilson et al., 2004) to drive expression in a subpopulation of olfactory projection neurons (PNs) in the antennal lobe, a key olfactory relay. Immunohistochemistry reveals strong CD4 staining, localized to the antennal lobe in transgenic GH146-GAL4 > HaloTag-CD4 flies (Figures 5f–h). These neurons also showed good extracellular staining (Figures 5i–l).

**FIGURE 6:**
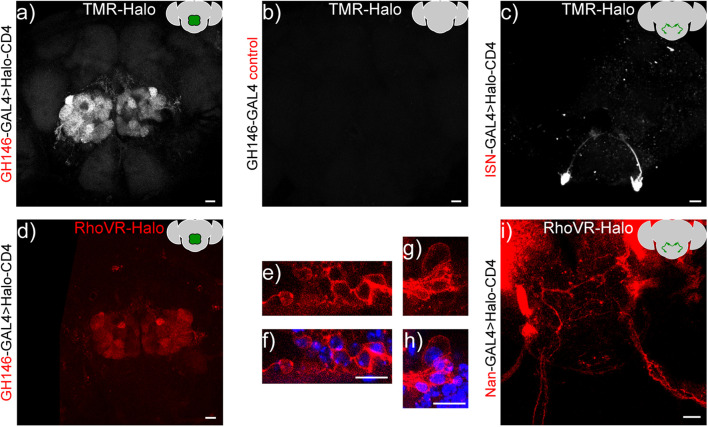
*In vivo* labeling of *Drosophila* neurons with TMR-Halo or RhoVR-Halo. Top row: Maximum z-projection of a confocal fluorescence microscopy stack of live brain explants labeled with voltage-*insensitive* TMR-Halo (1 μM) in an intact, live-fly before dissection and imaging. Crosses were either **(a)** GH146-GAL4 > HaloTag-CD4, **(b)** GH146-only control, or **(c)** VT011155-GAL4 > HaloTag-CD4. Bottom row. Maximum or sum z-projections of confocal fluorescence microscopy stack of live brain explants labeled with voltage-*sensitive* RhoVR-Halo (1–2 μM), labeling either **(d–h)** projection neurons (GH146-GAL4 > Halo-CD4, max projection) or **(I)** ISNs (Nan-GAL4 > Halo-CD4, sum projection). **(e,g)** High magnification images of RhoVR-Halo staining in PNs (red), overlaid with **(f,h)** Hoechst 33342 nuclear stain (blue). All scale bars are 20 μm.

HaloTag remains functional when expressed on the cell surface of *Drosophila* neurons, enabling a range of brain regions and neurons to be labeled with small molecules. We delivered TMR-Halo (1 μM) to live flies *via* application of a solution of TMR-Halo in artificial hemolymph (AHL) to flies with their cuticle removed (Harris et al., 2015) (see Supplementary Material for dissection details). We then imaged *via* confocal microscopy to establish the extent of labeling (Figure 6). In GH146-GAL4 > HaloTag flies (PN labeling) treated with TMR-Halo, we observe strong fluorescence localized to the antennal lobe (Figure 6a). Non-transgenic fly controls show low fluorescence levels in the brain and antennal lobe (GH146-GAL alone, Figure 6b). TMR-Halo in combination with HaloTag-CD4 can be used to label single cells. VT011155-GAL4 > HaloTag-CD4 fly brains drive expression in single interoceptive subesophageal zone neurons (ISNs) (Jourjine et al., 2016), and treatment with TMR-Halo results in bright fluorescence localized to these neurons (Figure 6c). Similar staining profiles can be achieved with the voltage-sensitive RhoVR-Halo, which clearly labels PNs of the antennal lobe (Figure 6d, GH146-GAL4). High magnification examination of labeled projection neurons reveals membrane-localized staining (Figures 6e,g, red) alongside Hoechst 33342 nuclear staining (Figures 6f,h, blue). RhoVR-Halo can also label smaller sub-sets of neurons cells; treatment of Nan-GAL4 > HaloTag-CD4 brains with RhoVR-Halo results in labeling of ISNs (Figure 6i).

We used the same live-animal staining procedure to optimize the loading of RhoVR-Halo (Supplementary Figure 5). We find that 2 μM RhoVR-Halo provides good staining in the antenna lobes of GH146-GAL4 > HaloTag-CD4 crosses (Supplementary Figure 5). Fluorescence from RhoVR-Halo is localized to the periphery of cell bodies, again supporting the extracellular expression of HaloTag-CD4 (Figures 6e–h). Compared to regions of the brain that do not express HaloTag-CD4, RhoVR-Halo fluorescence is approximately three times higher (Supplementary Figures 5B,C). We find homozygous flies for GH146-GAL4 > HaloTag-CD4 have slightly higher fluorescence levels compared to levels of heterozygous flies, when stained with the same concentration of RhoVR-Halo (Supplementary Figures 5H–J). However, because the difference in fluorescence intensity in homozygous flies was not significantly larger than heterozygotes, we used heterozygous flies for subsequent experiments.

### Functional Imaging

**FIGURE 7:**
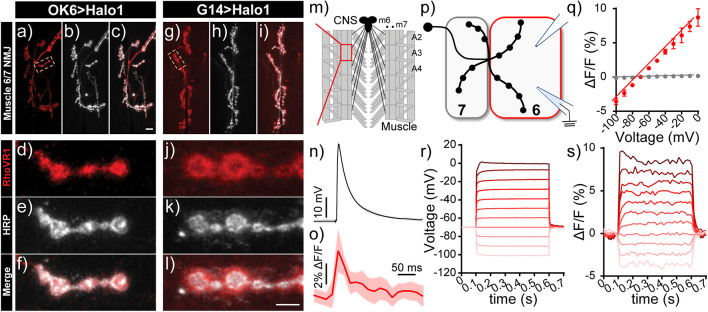
Voltage imaging with RhoVR-Halo using the *Drosophila* neuromuscular junction (NMJ). **(a–l)** Confocal images of motor neurons labeled with RhoVR-Halo (2 μM) in NMJs of **(a–f)** presynaptic neuron-labeled OK6-GAL4 > Halo-CD4 flies or **(g–l)** post-synaptic muscle-labeled G14-GAL4 > Halo-CD4 flies. Red is RhoVR-Halo fluorescence; gray is HRP—a neuronal membrane marker. Scale bars are 10 μm **(a–c,g–i)** and 5 μm **(d–f,j–l)**. **(M)** Schematic of *Drosophila* NMJ. Excitatory post-synaptic potentials (EPSPs) recorded at NMJs of G14-GAL4 > Halo-CD4 larvae stained with RhoVR-Halo (2 μM). Sharp electrode recordings of EPSPs are in **(n)** gray, and **(o)** optically recorded EPSPs are in red. Data are mean ± SEM of eight replicates. **(p)** Schematic of two-electrode measurements. Muscle cell 7 is unclamped, while the membrane potential of muscle cell 6 (m6) is clamped, held at –70 mV, and stepped to hyper- and depolarizing potentials ranging from –100 mV to 0 mV. **(q)** Plot of ΔF/F vs. holding potential for m6 (clamped, red) or m7 (unclamped, gray) in G14-GAL4 > Halo-CD4 flies stained with RhoVR-Halo. Data are mean ± standard error of the mean for *n* = 8 independent determinations. Example plots of change in **(r)** voltage or **(s)** fluorescence (ΔF/F) vs. time for the clamped m6 cell.

We established the voltage sensitivity of RhoVR-Halo in fly tissue expressing HaloTag-CD4 using two different approaches. First, we performed electrophysiology using dual two-electrode voltage-clamp combined with fluorescence imaging at the larval *Drosophila* neuromuscular junction (NMJ). We used the motor neuron driver OK6-GAL4 to drive pre-synaptic expression of HaloTag-CD4 (Figures 7a–f) or the muscle driver G14-GAL4 to express HaloTag-CD4 in the post-synaptic muscle (Figures 7g–l). In live 3rd instar larval NMJ preparations, RhoVR-Halo (2 μM) clearly stains pre-synaptic neuronal compartments when HaloTag-CD4 expression is targeted in motor neurons (red, Figure 7d), co-localizing with the neuronal plasma membrane marker horseradish peroxidase (HRP, gray, Figures 7e,f). In a complementary fashion, when HaloTag-CD4 is expressed in post-synaptic muscle cells, RhoVR-Halo fluorescence (red, Figure 7j) accumulates at NMJs outside of the neuronal membrane outlined by HRP (gray, Figures 7k,l). RhoVR-Halo readily detects excitatory post-synaptic potentials (EPSPs) in muscle cells, confirmed by simultaneous optical imaging and sharp electrode recordings (Figures 7m–o). Importantly, we next used two-electrode voltage-clamp recordings in a semi-dissected larval preparation with muscle HaloTag-CD4 expression (G14-GAL4 > HaloTag-CD4, Figure 7p). This approach demonstrated that depolarizing potentials result in an increase in RhoVR-Halo signal (m6, Figures 7q–s) with an overall voltage sensitivity of approximately 12% ΔF/F per 100 mV (± 0.2%, *n* = 8), in reasonably close agreement to the value determined in HEK293T cells (14%, Figure 4). Analysis of electrode (Figure 7r) and optical recordings (Figure 7s) show good correspondence. In contrast, no change in fluorescence signals was observed in an adjacent unclamped/unstimulated muscle cell (m7, Figure 7q, gray).

As a second confirmation of voltage sensitivity in fly tissues, we developed a stereotyped stimulation protocol for imaging in fly brain explants. We generated flies that express both HaloTag and the voltage-sensitive fluorescent protein, ArcLight, in PNs (GH146 GAL4, HaloTag/CyO; ArcLight/TM2) for use as an internal positive control. The use of RhoVR-Halo, with excitation and emission profiles in the green/orange region of the visible spectrum, allows for the simultaneous deployment of GFP-based indicators (Deal et al., 2016, 2020), like ArcLight (Jin et al., 2012). *Drosophila* antennal lobe projection neurons receive input from the olfactory receptor neurons (ORNs) in the antennae (Kazama and Wilson, 2008). As these projection neurons primarily receive cholinergic input from the ORNs (Restifo and White, 1990), we hypothesized that PNs could be readily stimulated with carbachol (CCH), a non-hydrolyzable acetylcholine mimic. We treated ArcLight/HaloTag-CD4 expressing fly brain explants with carbachol (100 μM) and observed robust fluorescence decreases timed to carbachol treatment, indicating a depolarizing membrane potential response to this neurotransmitter analog (Supplementary Figures 6A–D).

**FIGURE 8:**
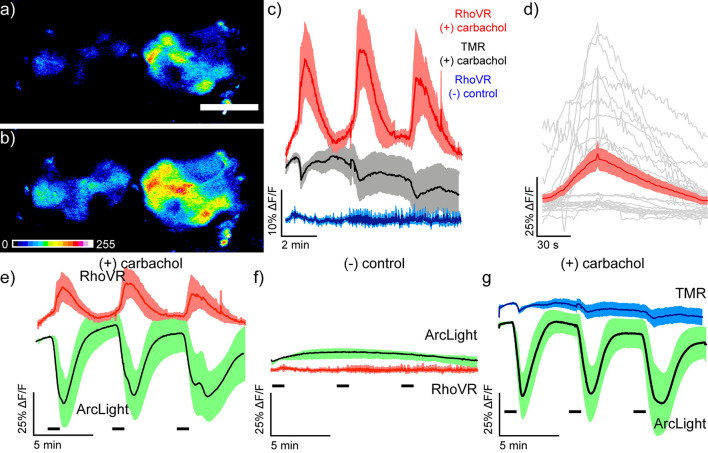
Simultaneous two-color visualization of carbachol-induced depolarization in projection neurons of live *Drosophila* brain explants with RhoVR-Halo and ArcLight. Epifluorescence images of live explant *Drosophila* brain expressing HaloTag-CD4 in antennal lobe projection neurons (GH146-GAL4, UAS-HaloTag-CD4/CyO; UAS-ArcLight/TM2) and labeled with RhoVR-Halo (2 μM) in live flies before dissection and explant imaging **(a)** immediately before and **(b)** 30 s after stimulation with 100 μM carbachol. Scale bar is 50 μm. **(c)** Plots of average ΔF/F traces for *Drosophila* brains under the following conditions: stained with voltage-sensitive RhoVR-Halo (2 μM) and stimulated with 100 μM carbachol (red, *n* = 7 brains), stained with voltage-insensitive TMR-Halo (100 nM) and stimulated with 100 μM carbachol (gray, *n* = 7 brains), or stained with voltage-sensitive RhoVR-Halo (2 μM) and treated with vehicle control (blue, *n* = 6 brains). **(d)** Plots of individual ΔF/F responses from RhoVR-Halo to carbachol stimuli (gray) and the average across all responses (red, SEM in light red). Traces of responses were aligned by peak response time and display 50 s before peak response and 150 s after peak response (gray). **(e)** Plots of average ΔF/F traces for *Drosophila* brains stained with voltage-sensitive RhoVR-Halo (2 μM) and stimulated with 100 μM carbachol (red, *n* = 7 brains). ArcLight responses are recorded simultaneously (green, *n* = 7 brains). RhoVR traces are replicated from panel **(c)** for comparison with ArcLight. **(f)** Plots of average ΔF/F traces for *Drosophila* brains stained with voltage-sensitive RhoVR-Halo (2 μM) and then treated with a vehicle control (red, *n* = 6 brains). ArcLight responses are recorded simultaneously (green, *n* = 6 brains). RhoVR traces are replicated from panel **(c)** for comparison with ArcLight. **(g)** Plots of average ΔF/F traces for *Drosophila* brains stained with voltage-*insensitive* TMR-Halo (100 nM) and stimulated with 100 μM carbachol (blue, *n* = 6 brains). ArcLight responses are recorded simultaneously (green, *n* = 6 brains). TMR traces are replicated from panel **(c)** for comparison with ArcLight. For all plots, data are mean ± SEM for the indicated number of samples. *Drosophila* brain explants were stimulated three times for 30 s with either 100 μM carbachol or vehicle. Stimulus (delivery of carbachol or vehicle) is depicted by small black bars immediately below the traces).

Using this robust stimulation protocol in fly brain explants, we next performed two-color voltage imaging with RhoVR-Halo and ArcLight. As before, we loaded RhoVR-Halo (2 μM) in live flies, removed the brains, and imaged the brain explants using epifluorescence microscopy. Excitation provided alternately with blue (475 nm) or green (542 nm) light to excite ArcLight or RhoVR-Halo, respectively, revealed robust fluorescence responses to carbachol (100 μM) treatment (Figure 8). RhoVR-Halo fluorescence increases with carbachol stimulation (Figures 8a–d), corresponding to membrane voltage depolarization and the turn-on response of RhoVR-type indicators (Deal et al., 2016, 2020). In contrast, ArcLight fluorescence decreases with carbachol stimulation (Figure 8e), showing a fluorescence decrease in response to depolarization, consistent with the turn-off response to depolarization for ArcLight indicators (Jin et al., 2012). Importantly, neither RhoVR-Halo nor ArcLight responds to a control experiment that omits carbachol from the perfusion solution (Figures 8c,f). Finally, the chemical-genetic hybrid approach of RhoVR-Halo enables additional controls to be carried out using the same transgenic flies. When HaloTag/ArcLight expressing flies are treated with TMR-Halo and then stimulated with carbachol, there is no response from the voltage-insensitive TMR-Halo (Figures 8c,g), but ArcLight still responds (Figure 8g). Using a “functionally dead” rhodamine dye in this experiment allows for control experiments to be run in the same transgenic animals as the experiments. Similar experiments with inactive mutants of genetically-encoded indicators/actuators (like ArcLight or GCaMP) would require the generation of separate transgenic animals with the inactivating mutation.

**FIGURE 9:**
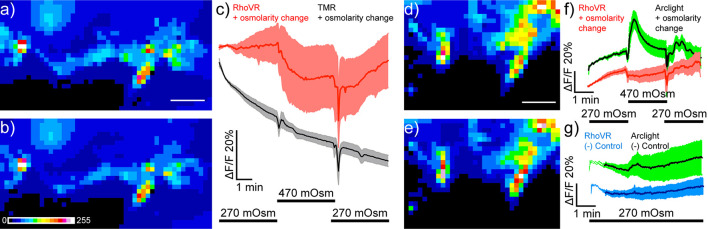
Imaging osmolarity induced hyperpolarizations in *Drosophila* interoceptive neurons in live explants using single color and dual-color imaging. Spinning disk confocal maximum z-projections of live explant *Drosophila* brain expressing HaloTag in ISNs (Nanchung-Gal4, UAS-HaloTag/Nanchung-Gal4, UAS-HaloTag; TM2/TM6B) **(a)** before and **(b)** after stimulation with high osmolarity hemolymph (470 mOsm). Scale bar is 50 μm. Image is pseudo-colored, and the scale bar indicates 8-bit pixel gray values. **(c)** Plot mean of fluorescence (%ΔF/F) vs. time in response to one osmolarity simulation of either RhoVR-Halo (red, *n* = 5) or HT-TMR (black, *n* = 5) loaded brains. Data are mean ± SEM Black bars below indicate the stimulation time course switching from 270 to 470 mOsm. Maximum z-projections of live explant *Drosophila* brain expressing HaloTag and Arclight in the ISNs (Nanchung-Gal4, UAS-HaloTag/UAS-Arclight; TM2/TM6B) **(d)** before and **(e)** after stimulation with high osmolarity hemolymph (470 mOsm). **(f)** Plot mean of fluorescence (%ΔF/F) vs. time for simultaneously imaged Arclight (green) and RhoVR-Halo (red) in response to high osmolarity simulation (*n* = 7). **(g)** Plot mean of fluorescence (%ΔF/F) vs. time for simultaneously imaged Arclight (green) and RhoVR-Halo (red) in response a vehicle control (*n* = 7).

To evaluate the ability of RhoVR-Halo to report on physiological stimuli, we probed the response of RhoVR-Halo in ISNs, cells that respond dynamically to changes in osmolarity. Previous studies demonstrated that increases in osmolarity (240–440 mOsm) evoke hyperpolarizing responses in ISNs (Jourjine et al., 2016). Consistent with this, we find that ISNs expressing HaloTag-CD4 (Nanchung-GAL4) and labeled with RhoVR-Halo hyperpolarize upon an increase in osmolarity, as indicated by decreases in RhoVR fluorescence (Figures 9a–c). In fly brains labeled with voltage-insensitive TMR-Halo, we observe no change in fluorescence (Figures 9a–c). In contrast, flies expressing ArcLight in ISNs show fluorescence increases in response to increased osmolarity (Supplementary Figure 7). Two-color voltage imaging alongside ArcLight in flies that express both HaloTag-CD4 and ArcLight in ISNs (Nanchung-GAL4, UAS-HaloTag-CD4/CyO; UAS-ArcLight/TM2) reveals osmolarity-induced decreases in RhoVR fluorescence coupled with increases in ArcLight fluorescence (Figures 9d–f), while control experiments at constant osmolarity show no responses in either ArcLight or RhoVR fluorescence (Figure 9g). Heterozygous flies expressing HaloTag in ISNs and labeled with RhoVR-Halo also respond to changes in osmolarity (Supplementary Figure 8). Taken together, these data establish the utility of RhoVR-Halo for monitoring sensory-induced changes to membrane potential.

## Discussion

In summary, we show that RhoVR-Halo indicators can be used for direct visualization of membrane potential changes in synapses and brains of flies. We show, for the first time, that RhoVR-Halo dyes can label specific neurons *in vivo* and that voltage changes can be visualized using epifluorescence microscopy at synapses in the NMJ and whole-brain explants. The hybrid chemical-genetic strategy employed here features a turn-on response to membrane depolarization and affords the opportunity to “plug-and-play” different fluorescent dyes to enable imaging in different colors (Ortiz et al., 2020) or to run critical control experiments using a non-voltage-sensitive fluorophore in the same genetic background (Figures 8g–i). We envision that RhoVR-Halos, with their high two-photon (2P) cross-section (93 GM at 840 nm, Supplementary Figure 9), can be combined with high-speed 2P imaging methods to provide fast voltage imaging in the brain.

Despite these advances, several drawbacks are associated with our implementation of the methodology at present. First, in the imaging data presented here, we do not take full advantage of the response kinetics of PeT-based indicators like RhoVR, which should have nanosecond responses times based on the mechanism of voltage sensing (Beier et al., 2019; Lazzari-Dean et al., 2019; Milosevic et al., 2020). Secondly, we do not take full advantage of the high 2P excitation cross-section of RhoVR dyes. Especially notable is the substantial cross-section at ∼1,030–1,040^13^ nm (Supplementary Figure 9), which allows for the use of high-powered 2P illumination in emerging fast 2P methods (Kazemipour et al., 2019; Wu et al., 2020). Third, in fly brains, RhoVR-Halo voltage-sensitive indicators are not as bright as their fluorophore-only counterparts. This is likely a result of combinations of (a) lower intrinsic quantum yield for RhoVR-Halo compared to TMR-Halo (since the presence of a molecular wire quenches the fluorescence of the dye) and (b) lower solubility for the rather greasy RhoVR-Halo indicators compared to the smaller, more compact TMR-Halo dyes. The former can be addressed by using published methods to generate brighter fluorophores. The latter can be addressed by the use of new chemistries to attach HaloTag ligands, freeing up other sites for solubilizing groups to address the challenge of delivering fluorophores to intact brain preparations. Even with these limitations, we envision that chemical-genetic hybrids like RhoVR-Halo will be an important complement to the expanding set of methods for visualizing membrane potential changes in living systems.

## Data Availability Statement

The raw data supporting the conclusions of this article will be made available by the authors, without undue reservation.

## Author Contributions

MK, BB, YH, MD, DD, KS, and EM designed the experiments. MK, BB, YH, AR, AG, RM, and MD performed the experiments. PD, MD, MK, and BB contributed unpublished reagents and analysis. MK, BB, YH, RM, MD, DD, KS, and EM analyzed data. MK, BB, YH, DD, KS, and EM wrote manuscript. All authors contributed to the article and approved the submitted version.

## Conflict of Interest

The authors declare that the research was conducted in the absence of any commercial or financial relationships that could be construed as a potential conflict of interest.

## Publisher’s Note

All claims expressed in this article are solely those of the authors and do not necessarily represent those of their affiliated organizations, or those of the publisher, the editors and the reviewers. Any product that may be evaluated in this article, or claim that may be made by its manufacturer, is not guaranteed or endorsed by the publisher.
