# Supplementary material for: Voltage Imaging in Drosophila Using a Hybrid Chemical-Genetic Rhodamine Voltage Reporter
Source: Front Neurosci. 2021 Nov 16;15:754027. doi: 10.3389/fnins.2021.754027 (PMC8637050; doi:10.3389/fnins.2021.754027)
Supplement: Supplementary file 1 [file Table_1.DOCX]

LOCUS pJFRC7-PAT3-Halo(V2)-_C 9839 bp ds-DNA circular 03-JUN-2021

DEFINITION .

FEATURES Location/Qualifiers

misc_feature 706..990

/label="AttB"

/ApEinfo_revcolor=#ff9ccd

/ApEinfo_fwdcolor=#ff9ccd

misc_feature 1840..4494

/label="mini white NGS"

/ApEinfo_revcolor=#f58a5e

/ApEinfo_fwdcolor=#f58a5e

misc_feature 5140..5925

/label="20xUAS"

/ApEinfo_revcolor=#85dae9

/ApEinfo_fwdcolor=#85dae9

misc_feature 5894..6121

/label="HS promoter"

/ApEinfo_revcolor=#faac61

/ApEinfo_fwdcolor=#faac61

primer 5905..5925

/label="Tims UAS geno F1"

/note="sequence: AAATAGAGGCGCTTCGTCTAC"

/ApEinfo_revcolor=#ff9ccd

/ApEinfo_fwdcolor=#ff9ccd

primer 6204..6240

/label="Pat3 PJFRC7 Gib F1 RE Frag"

/note="sequence: CCTTTACTTCAGGCGGCCGCGGCTCGAGATGCCACCT"

/ApEinfo_revcolor=#f8d3a9

/ApEinfo_fwdcolor=#f8d3a9

primer 6204..6249

/label="PJFRC7 Pat3 Gib 12/11"

/note="sequence: CCTTTACTTCAGGCGGCCGCGGCTCGAGATGCCACCTTCAACATCA"

/ApEinfo_revcolor=#c6c9d1

/ApEinfo_fwdcolor=#c6c9d1

primer 6206..6249

/label="PJFR7 Pat3 Gib 12/11 2"

/note="sequence: TTTACTTCAGGCGGCCGCGGCTCGAGATGCCACCTTCAACATCA"

/ApEinfo_revcolor=#b1ff67

/ApEinfo_fwdcolor=#b1ff67

primer 6212..6249

/label="PJFRC7 Pat3 GIB 2/24"

/note="sequence: TCAGGCGGCCGCGGCTCGAGATGCCACCTTCAACATCA"

/ApEinfo_revcolor=#faac61

/ApEinfo_fwdcolor=#faac61

misc_feature 6232..6319

/label="Pat-3 "

/ApEinfo_revcolor=#9eafd2

/ApEinfo_fwdcolor=#9eafd2

CDS 6232..6324

/label="Translation 6232-6324"

misc_feature 6325..7212

/label="HaloTag"

/ApEinfo_revcolor=#c7b0e3

/ApEinfo_fwdcolor=#c7b0e3

CDS 6325..7212

/label="Translation 6325-7212"

primer complement(6385..6403)

/label="Halo Genotyping R2"

/note="sequence: CAACATCGACGTAGTGCAT"

/ApEinfo_revcolor=#faac61

/ApEinfo_fwdcolor=#faac61

primer 6569..6583

/label="Halo Mid Seq F2"

/note="sequence: ACCACGTCCGCTTCA"

/ApEinfo_revcolor=#faac61

/ApEinfo_fwdcolor=#faac61

misc_feature 7213..7242

/label="Linker"

/ApEinfo_revcolor=#85dae9

/ApEinfo_fwdcolor=#85dae9

CDS 7213..7923

/label="Translation 7213-7923"

primer complement(7232..7257)

/label="Halo CD4 R2 NoTag"

/note="sequence: CTTCTGGAAGTCGACCGAGCCTCCAC"

/ApEinfo_revcolor=#c6c9d1

/ApEinfo_fwdcolor=#c6c9d1

misc_feature 7249..7923

/label="CD4 "

/ApEinfo_revcolor=#ff9ccd

/ApEinfo_fwdcolor=#ff9ccd

primer 7449..7466

/label="CD45'seq"

/note="sequence: TAAGCTCCAGATGGGCAA"

/ApEinfo_revcolor=#d6b295

/ApEinfo_fwdcolor=#d6b295

primer complement(7912..7928)

/label="pJFRC7 CD4 Gib from RE frag"

/note="sequence: CTAGACTAGCGCCTTCG"

/ApEinfo_revcolor=#b7e6d7

/ApEinfo_fwdcolor=#b7e6d7

primer complement(7912..7946)

/label="PJFRC7 CD4 Gib 2/24"

/note="sequence: TTCCTTCACAAAGATCCTCTAGACTAGCGCCTTCG"

/ApEinfo_revcolor=#f58a5e

/ApEinfo_fwdcolor=#f58a5e

primer complement(7912..7949)

/label="PJFRC7 CD4 gib 12/11"

/note="sequence: AGGTTCCTTCACAAAGATCCTCTAGACTAGCGCCTTCG"

/ApEinfo_revcolor=#ff9ccd

/ApEinfo_fwdcolor=#ff9ccd

primer complement(7913..7949)

/label="pJFRC7 CD4 Gib from RE frag"

/note="sequence: AGGTTCCTTCACAAAGATCCTCTAGACTAGCGCCTTC"

/ApEinfo_revcolor=#d59687

/ApEinfo_fwdcolor=#d59687

misc_feature 7924..7924

/label="mCD8GFP"

/ApEinfo_revcolor=#d6b295

/ApEinfo_fwdcolor=#d6b295

misc_feature 7925..8432

/label="WPRE Ect. "

/ApEinfo_revcolor=#faac61

/ApEinfo_fwdcolor=#faac61

polyA_signal complement(8433..8624)

/label="SV40 late polyA"

/ApEinfo_revcolor=#c6c9d1

/ApEinfo_fwdcolor=#c6c9d1

CDS 9058..9717

/label="AmpR"

/ApEinfo_revcolor=#ffef86

/ApEinfo_fwdcolor=#ffef86

ORIGIN

1 GTTTTCGTTC CACTGAGCGT CAGACCCCGT AGAAAAGATC AAAGGATCTT CTTGAGATCC

61 TTTTTTTCTG CGCGTAATCT GCTGCTTGCA AACAAAAAAA CCACCGCTAC CAGCGGTGGT

121 TTGTTTGCCG GATCAAGAGC TACCAACTCT TTTTCCGAAG GTAACTGGCT TCAGCAGAGC

181 GCAGATACCA AATACTGTTC TTCTAGTGTA GCCGTAGTTA GGCCACCACT TCAAGAACTC

241 TGTAGCACCG CCTACATACC TCGCTCTGCT AATCCTGTTA CCAGTGGCTG CTGCCAGTGG

301 CGATAAGTCG TGTCTTACCG GGTTGGACTC AAGACGATAG TTACCGGATA AGGCGCAGCG

361 GTCGGGCTGA ACGGGGGGTT CGTGCACACA GCCCAGCTTG GAGCGAACGA CCTACACCGA

421 ACTGAGATAC CTACAGCGTG AGCTATGAGA AAGCGCCACG CTTCCCGAAG GGAGAAAGGC

481 GGACAGGTAT CCGGTAAGCG GCAGGGTCGG AACAGGAGAG CGCACGAGGG AGCTTCCAGG

541 GGGAAACGCC TGGTATCTTT ATAGTCCTGT CGGGTTTCGC CACCTCTGAC TTGAGCGTCG

601 ATTTTTGTGA TGCTCGTCAG GGGGGCGGAG CCTATGGAAA AACGCCAGCA ACGCGGCCTT

661 TTTACGGTTC CTGGCCTTTT GCTGGCCTTT TGCTCACATG TTACCGTCGA CGATGTAGGT

721 CACGGTCTCG AAGCCGCGGT GCGGGTGCCA GGGCGTGCCC TTGGGCTCCC CGGGCGCGTA

781 CTCCACCTCA CCCATCTGGT CCATCATGAT GAACGGGTCG AGGTGGCGGT AGTTGATCCC

841 GGCGAACGCG CGGCGCACCG GGAAGCCCTC GCCCTCGAAA CCGCTGGGCG CGGTGGTCAC

901 GGTGAGCACG GGACGTGCGA CGGCGTCGGC GGGTGCGGAT ACGCGGGGCA GCGTCAGCGG

961 GTTCTCGACG GTCACGGCGG GCATGTCGAC AAGCCGAACA TATGGGCGCG CCTAGTATGT

1021 ATGTAAGTTA ATAAAACCCA TTTTTGCGGA AAGTAGATAA AAAAAACATT TTTTTTTTTT

1081 ACTGCACTGG ATATCATTGA ACTTATCTGA TCAGTTTTAA ATTTACTTCG ATCCAAGGGT

1141 ATTTGATGTA CCAGGTTCTT TCGATTACCT CTCACTCAAA ATGACATTCC ACTCAAAGTC

1201 AGCGCTGTTT GCCTCCTTCT CTGTCCACAG AAATATCGCC GTCTCTTTCG CCGCTGCGTC

1261 CGCTATCTCT TTCGCCACCG TTTGTAGCGT TACGTAGCGT CAATGTCCGC CTTCAGTTGC

1321 ATTTTGTCAG CGGTTTCGTG ACGAAGCTCC AAGCGGTTTA CGCCATCAAT TAAACACAAA

1381 GTGCTGTGCC AAAACTCCTC TCGCTTCTTA TTTTTGTTTG TTTTTTGAGT GATTGGGGTG

1441 GTGATTGGTT TTGGGTGGGT AAGCAGGGGA AAGTGTGAAA AATCCCGGCA ATGGGCCAAG

1501 AGGATCAGGA GCTATTAATT CGCGGAGGCA GCAAACACCC ATCTGCCGAG CATCTGAACA

1561 ATGTGAGTAG TACATGTGCA TACATCTTAA GTTCACTTGA TCTATAGGAA CTGCGATTGC

1621 AACATCAAAT TGTCTGCGGC GTGAGAACTG CGACCCACAA AAATCCCAAA CCGCAATTGC

1681 ACAAACAAAT AGTGACACGA AACAGATTAT TCTGGTAGCT GTTCTCGCTA TATAAGACAA

1741 TTTTTGAGAT CATATCATGA TCAAGACATC TAAAGGCATT CATTTTCGAC TATATTCTTT

1801 TTTACAAAAA ATATAACAAC CAGATATTTT AAGCTGATCC TAGATGCACA AAAAATAAAT

1861 AAAAGTATAA ACCTACTTCG TAGGATACTT CGGGGTACTT TTTGTTCGGG GTTAGATGAG

1921 CATAACGCTT GTAGTTGATA TTTGAGATCC CCTATCATTG CAGGGTGACA GCGGAGCGGC

1981 TTCGCAGAGC TGCATTAACC AGGGCTTCGG GCAGGCCAAA AACTACGGCA CGCTCCGGCC

2041 ACCCAGTCCG CCGGAGGACT CCGGTTCAGG GAGCGGCCAA CTAGCCGAGA ACCTCACCTA

2101 TGCCTGGCAC AATATGGACA TCTTTGGGGC GGTCAATCAG CCGGGCTCCG GATGGCGGCA

2161 GCTGGTCAAC CGGACACGCG GACTATTCTG CAACGAGCGA CACATACCGG CGCCCAGGAA

2221 ACATTTGCTC AAGAACGGTG AGTTTCTATT CGCAGTCGGC TGATCTGTGT GAAATCTTAA

2281 TAAAGGGTCC AATTACCAAT TTGAAACTCA GTTTGCGGCG TGGCCTATCC GGGCGAACTT

2341 TTGGCCGTGA TGGGCAGTTC CGGTGCCGGA AAGACGACCC TGCTGAATGC CCTTGCCTTT

2401 CGATCGCCGC AGGGCATCCA AGTATCGCCA TCCGGGATGC GACTGCTCAA TGGCCAACCT

2461 GTGGACGCCA AGGAGATGCA GGCCAGGTGC GCCTATGTCC AGCAGGATGA CCTCTTTATC

2521 GGCTCCCTAA CGGCCAGGGA ACACCTGATT TTCCAAGCCA TGGTGCGGAT GCCACGACAT

2581 CTGACCTATC GGCAGCGAGT GGCCCGCGTG GATCAGGTGA TCCAGGAGCT TTCGCTCAGC

2641 AAATGTCAGC ACACGATCAT CGGTGTGCCC GGCAGGGTGA AAGGTCTGTC CGGCGGAGAA

2701 AGGAAGCGTC TGGCATTCGC CTCCGAGGCT CTAACCGATC CGCCGCTTCT GATCTGCGAT

2761 GAGCCCACCT CCGGACTGGA CTCCTTTACC GCCCACAGCG TCGTCCAGGT GCTGAAGAAG

2821 CTGTCGCAGA AGGGCAAGAC CGTCATCCTG ACCATTCATC AGCCGTCTTC CGAGCTGTTT

2881 GAGCTCTTTG ACAAGATCCT TCTGATGGCC GAGGGCAGGG TAGCTTTCTT GGGCACTCCC

2941 AGCGAAGCCG TCGACTTCTT TTCCTAGTGA GTTCGATGTG TTTATTAAGG GTATCTAGTA

3001 TTACATAACA TCTCAACTCC TATCCAGCGT GGGTGCCCAG TGTCCTACCA ACTACAATCC

3061 GGCGGACTTT TACGTACAGG TGTTGGCCGT TGTGCCCGGA CGGGAGATCG AGTCCCGTGA

3121 TCGGATCGCC AAGATATGCG ACAATTTTGC CATTAGCAAA GTAGCCCGGG ATATGGAGCA

3181 GTTGTTGGCC ACCAAAAATC TGGAGAAGCC ACTGGAGCAG CCGGAGAATG GGTACACCTA

3241 CAAGGCCACC TGGTTCATGC AGTTCCGGGC GGTCCTGTGG CGATCCTGGC TGTCGGTGCT

3301 CAAGGAACCA CTCCTCGTAA AAGTGCGACT TATTCAGACA ACGGTGAGTG GTTCCAGTGG

3361 AAACAAATGA TATAACGCTT ACAATTCTTG GAAACAAATT CGCTAGATTT TAGATAGAAT

3421 TGCCTGATTC CACACCCTTC TTAGTTTTTT TCAATGAGAT GTATAGTTTA TAGTTTTGCA

3481 GAAGATAAAT AAATTTCATT TAACTCGCGA ATATTAATGA GATGCGAGTA ACATTTTAAT

3541 TTGCAGATGG TTGCCATCTT GATTGGCCTC ATCTTTTTGG GCCAACAACT CACGCAAGTG

3601 GGTGTGATGA ATATCAACGG AGCCATCTTC CTCTTCCTGA CCAACATGAC CTTTCAAAAC

3661 GTCTTTGCCA CGATAAATGT AAGTCATGTT TAGAATACAT TTGCATTTCA ATAATTTACT

3721 AACTTTCTAA TGAATCGATT CGATTTAGGT GTTCACCTCA GAGCTGCCAG TTTTTATGAG

3781 GGAGGCCCGA AGTCGACTTT ATCGCTGTGA CACATACTTT CTGGGCAAAA CGATTGCCGA

3841 ATTGCCGCTT TTTCTCACAG TGCCACTGGT CTTCACGGCG ATTGCCTATC CGATGATCGG

3901 ACTGCGGGCC GGAGTGCTGC ACTTCTTCAA CTGCCTGGCG CTGGTCACTC TGGTGGCCAA

3961 TGTGTCAACG TCCTTCGGAT ATCTAATATC CTGCGCCAGC TCCTCGACCT CGATGGCGCT

4021 GTCTGTGGGT CCGCCGGTTA TCATACCATT CCTGCTCTTT GGCGGCTTCT TCTTGAACTC

4081 GGGCTCGGTG CCAGTATACC TCAAATGGTT GTCGTACCTC TCATGGTTCC GTTACGCCAA

4141 CGAGGGTCTG CTGATTAACC AATGGGCGGA CGTGGAGCCG GGCGAAATTA GCTGCACATC

4201 GTCGAACACC ACGTGCCCCA GTTCGGGCAA GGTCATCCTG GAGACGCTTA ACTTCTCCGC

4261 CGCCGATCTG CCGCTGGACT ACGTGGGTCT GGCCATTCTC ATCGTGAGCT TCCGGGTGCT

4321 CGCATATCTG GCTCTAAGAC TTCGGGCCCG ACGCAAGGAG TAGCCGACAT ATATCCGAAA

4381 TAACTGCTTG TTTTTTTTTT TTACCATTAT TACCATCGTG TTTACTGTTT ATTGCCCCCT

4441 CAAAAAGCTA ATGTAATTAT ATTTGTGCCA ATAAAAACAA GATATGACCT ATAGAATACA

4501 AGTATTTCCC CTTCGAACAT CCCCACAAGT AGACTTTGGA TTTGTCTTCT AACCAAAAGA

4561 CTTACACACC TGCATACCTT ACATCAAAAA CTCGTTTATC GCTACATAAA ACACCGGGAT

4621 ATATTTTTTA TATACATACT TTTCAAATCG CGCGCCCTCT TCATAATTCA CCTCCACCAC

4681 ACCACGTTTC GTAGTTGCTC TTTCGCTGTC TCCCACCCGC TCTCCGCAAC ACATTCACCT

4741 TTTGTTCGAC GACCTTGGAG CGACTGTCGT TAGTTCCGCG CGATTCGGTT CGCTCAAATG

4801 GTTCCGAGTG GTTCATTTCG TCTCAATAGA AATTAGTAAT AAATATTTGT ATGTACAATT

4861 TATTTGCTCC AATATATTTG TATATATTTC CCTCACAGCT ATATTTATTC TAATTTAATA

4921 TTATGACTTT TTAAGGTAAT TTTTTGTGAC CTGTTCGGAG TGATTAGCGT TACAATTTGA

4981 ACTGAAAGTG ACATCCAGTG TTTGTTCCTT GTGTAGATGC ATCTCAAAAA AATGGTGGGC

5041 ATAATAGTGT TGTTTATATA TATCAAAAAT AACAACTATA ATAATAAGAA TACATTTAAT

5101 TTAGAAAATG CTTGGATTTC ACTGGAACTA GGGCGCGCCT CCGGAACATA ATGGTGCAGG

5161 GCGCTGACTT CCGCGTTTCC AGACTTTACG AAACACGGAA ACCGAAGACC ATTCATGTTG

5221 TTGCTCAGGT CGCAGACGTT TTGCAGCAGC AGTCGCTTCA CGTTCGCTCG CGTATCGGTG

5281 ATTCATTCTG CTAACCAGTA AGGCAACCCC GCCAGCCTAG CCGGGTCCTC AACGACAGGA

5341 GCACGATCAT GCGCACCCGT GGCCAGGGCC GCAAGCTTGC ATGCCTGCAG GTCGGAGTAC

5401 TGTCCTCCGA GCGGAGTACT GTCCTCCGAG CGGAGTACTG TCCTCCGAGC GGAGTACTGT

5461 CCTCCGAGCG GAGTACTGTC CTCCGAGCGG AGACTCTAGC CCTAGGGCAT GCCTGCAGGT

5521 CGGAGTACTG TCCTCCGAGC GGAGTACTGT CCTCCGAGCG GAGTACTGTC CTCCGAGCGG

5581 AGTACTGTCC TCCGAGCGGA GTACTGTCCT CCGAGCGGAG ACTCTAGCGC TAGCGCATGC

5641 CTGCAGGTCG GAGTACTGTC CTCCGAGCGG AGTACTGTCC TCCGAGCGGA GTACTGTCCT

5701 CCGAGCGGAG TACTGTCCTC CGAGCGGAGT ACTGTCCTCC GAGCGGAGAC TCTAGCACTA

5761 GTGCATGCCT GCAGGTCGGA GTACTGTCCT CCGAGCGGAG TACTGTCCTC CGAGCGGAGT

5821 ACTGTCCTCC GAGCGGAGTA CTGTCCTCCG AGCGGAGTAC TGTCCTCCGA GCGGAGACTC

5881 TAGCGACGTC GAGCGCCGGA GTATAAATAG AGGCGCTTCG TCTACGGAGC GACAATTCAA

5941 TTCAAACAAG CAAAGTGAAC ACGTCGCTAA GCGAAAGCTA AGCAAATAAA CAAGCGCAGC

6001 TGAACAAGCT AAACAATCTG CAGTAAAGTG CAAGTTAAAG TGAATCAATT AAAAGTAACC

6061 AGCAACCAAG TAAATCAACT GCAACTACTG AAATCTGCCA AGAAGTAATT ATTGAATACA

6121 AGAAGAGAAC TCTGAATAGA TCTAAAAGGT AGGTTCAACC ACTGATGCCT AGGCACACCG

6181 AAACGACTAA CCCTAATTCT TATCCTTTAC TTCAGGCGGC CGCGGCTCGA GATGCCACCT

6241 TCAACATCAT TGCTGCTCCT CGCAGCACTT CTTCCATTCG CTTTACCAGC AAGCGATTGG

6301 AAGACTGGAG AAGTCACTGC TAGCGCAGAA ATCGGTACTG GCTTTCCATT CGACCCCCAT

6361 TATGTGGAAG TCCTGGGCGA GCGCATGCAC TACGTCGATG TTGGTCCGCG CGATGGCACC

6421 CCTGTGCTGT TCCTGCACGG TAACCCGACC TCCTCCTACG TGTGGCGCAA CATCATCCCG

6481 CATGTTGCAC CGACCCATCG CTGCATTGCT CCAGACCTGA TCGGTATGGG CAAATCCGAC

6541 AAACCAGACC TGGGTTATTT CTTCGACGAC CACGTCCGCT TCATGGATGC CTTCATCGAA

6601 GCCCTGGGTC TGGAAGAGGT CGTCCTGGTC ATTCACGACT GGGGCTCCGC TCTGGGTTTC

6661 CACTGGGCCA AGCGCAATCC AGAGCGCGTC AAAGGTATTG CATTTATGGA GTTCATCCGC

6721 CCTATCCCGA CCTGGGACGA ATGGCCAGAA TTTGCCCGCG AGACCTTCCA GGCCTTCCGC

6781 ACCACCGACG TCGGCCGCAA GCTGATCATC GATCAGAACG TTTTTATCGA GGGTACGCTG

6841 CCGATGGGTG TCGTCCGCCC GCTGACTGAA GTCGAGATGG ACCATTACCG CGAGCCGTTC

6901 CTGAATCCTG TTGACCGCGA GCCACTGTGG CGCTTCCCAA ACGAGCTGCC AATCGCCGGT

6961 GAGCCAGCGA ACATCGTCGC GCTGGTCGAA GAATACATGG ACTGGCTGCA CCAGTCCCCT

7021 GTCCCGAAGC TGCTGTTCTG GGGCACCCCA GGCGTTCTGA TCCCACCGGC CGAAGCCGCT

7081 CGCCTGGCCA AAAGCCTGCC TAACTGCAAG GCTGTGGACA TCGGCCCGGG TCTGAATCTG

7141 CTGCAAGAAG ACAACCCGGA CCTGATCGGC AGCGAGATCG CGCGCTGGCT GTCGACGCTC

7201 GAGATTTCCG GCGGTGGCGG CGGAAGTGGA GGTGGAGGCT CGGTCGACTT CCAGAAGGCC

7261 TCCAGCATAG TCTATAAGAA AGAGGGGGAA CAGGTGGAGT TCTCCTTCCC ACTCGCCTTT

7321 ACAGTTGAAA AGCTGACGGG CAGTGGCGAG CTGTGGTGGC AGGCGGAGAG GGCTTCCTCC

7381 TCCAAGTCTT GGATCACCTT TGACCTGAAG AACAAGGAAG TGTCTGTAAA ACGGGTTACC

7441 CAGGACCCTA AGCTCCAGAT GGGCAAGAAG CTCCCGCTCC ACCTCACCCT GCCCCAGGCC

7501 TTGCCTCAGT ATGCTGGCTC TGGAAACCTC ACCCTGGCCC TTGAAGCGAA AACAGGAAAG

7561 TTGCATCAGG AAGTGAACCT GGTGGTGATG AGAGCCACTC AGCTCCAGAA AAATTTGACC

7621 TGTGAGGTGT GGGGACCCAC CTCCCCTAAG CTGATGCTGA GCTTGAAACT GGAGAACAAG

7681 GAGGCAAAGG TCTCGAAGCG GGAGAAGGCG GTGTGGGTGC TGAACCCTGA GGCGGGGATG

7741 TGGCAGTGTC TGCTGAGTGA CTCGGGACAG GTCCTGCTGG AATCCAACAT CAAGGTTCTG

7801 CCCACATGGT CCACCCCGGT GCAGCCAATG GCCCTGATTG TGCTGGGGGG CGTCGCCGGC

7861 CTCCTGCTTT TCATTGGGCT AGGCATCTTC TTCTGTGTCA GGTGCCGGCA CCGAAGGCGC

7921 TAGTCTAGAG GATCTTTGTG AAGGAACCTT ACTTCTGTGG TGTGACATAA TTGGACAAAC

7981 TACCTACAGA GATTTAAAGC TCTAAGGTAA ATATAAAATT TTTAAGTGTA TAATGTGTTA

8041 AACTACTGAT TCTAATTGTT TGTGTATTTT AGATTCCAAC CTATGGAACT GATGAATGGG

8101 AGCAGTGGTG GAATGCCTTT AATGAGGAAA ACCTGTTTTG CTCAGAAGAA ATGCCATCTA

8161 GTGATGATGA GGCTACTGCT GACTCTCAAC ATTCTACTCC TCCAAAAAAG AAGAGAAAGG

8221 TAGAAGACCC CAAGGACTTT CCTTCAGAAT TGCTAAGTTT TTTGAGTCAT GCTGTGTTTA

8281 GTAATAGAAC TCTTGCTTGC TTTGCTATTT ACACCACAAA GGAAAAAGCT GCACTGCTAT

8341 ACAAGAAAAT TATGGAAAAA TATTTGATGT ATAGTGCCTT GACTAGAGAT CATAATCAGC

8401 CATACCACAT TTGTAGAGGT TTTACTTGCT TTAAAAAACC TCCCACACCT CCCCCTGAAC

8461 CTGAAACATA AAATGAATGC AATTGTTGTT GTTAACTTGT TTATTGCAGC TTATAATGGT

8521 TACAAATAAA GCAATAGCAT CACAAATTTC ACAAATAAAG CATTTTTTTC ACTGCATTCT

8581 AGTTGTGGTT TGTCCAAACT CATCAATGTA TCTTATCATG TCTGGATCGA TCTGGCCGGC

8641 CGTTTAAACG AATTCTTGAA GACGAAAGGG CCTCGTGATA CGCCTATTTT TATAGGTTAA

8701 TGTCATGATA ATAATGGTTT CTTAGACTCA GGTGGCACTT TTCGGGGAAA TGTGCGCGGA

8761 ACCCCTATTT GTTTATTTTT CTAAATACAT TCAAATATGT ATCCGCTCAT GAGACAATAA

8821 CCCTGATAAA TGCTTCAATA ATATTGAAAA AGGAAGAGTA TGAGTATTCA ACATTTCCGT

8881 GTCGCCCTTA TTCCCTTTTT TGCGGCATTT TGCCTTCCTG TTTTTGCTCA CCCAGAAACG

8941 CTGGTGAAAG TAAAAGATGC TGAAGATCAG TTGGGTGCAC GAGTGGGTTA CATCGAACTG

9001 GATCTCAACA GCGGTAAGAT CCTTGAGAGT TTTCGCCCCG AAGAACGTTT TCCAATGATG

9061 AGCACTTTTA AAGTTCTGCT ATGTGGCGCG GTATTATCCC GTATTGACGC CGGGCAAGAG

9121 CAACTCGGTC GCCGCATACA CTATTCTCAG AATGACTTGG TTGAGTACTC ACCAGTCACA

9181 GAAAAGCATC TTACGGATGG CATGACAGTA AGAGAATTAT GCAGTGCTGC CATAACCATG

9241 AGTGATAACA CTGCGGCCAA CTTACTTCTG ACAACGATCG GAGGACCGAA GGAGCTAACC

9301 GCTTTTTTGC ACAACATGGG GGATCATGTA ACTCGCCTTG ATCGTTGGGA ACCGGAGCTG

9361 AATGAAGCCA TACCAAACGA CGAGCGTGAC ACCACGATGC CTGTAGCAAT GGCAACAACG

9421 TTGCGCAAAC TATTAACTGG CGAACTACTT ACTCTAGCTT CCCGGCAACA ATTAATAGAC

9481 TGGATGGAGG CGGATAAAGT TGCAGGACCA CTTCTGCGCT CGGCCCTTCC GGCTGGCTGG

9541 TTTATTGCTG ATAAATCTGG AGCCGGTGAG CGTGGGTCTC GCGGTATCAT TGCAGCACTG

9601 GGGCCAGATG GTAAGCCCTC CCGTATCGTA GTTATCTACA CGACGGGGAG TCAGGCAACT

9661 ATGGATGAAC GAAATAGACA GATCGCTGAG ATAGGTGCCT CACTGATTAA GCATTGGTAA

9721 CTGTCAGACC AAGTTTACTC ATATATACTT TAGATTGATT TAAAACTTCA TTTTTAATTT

9781 AAAAGGATCT AGGTGAAGAT CCTTTTTGAT AATCTCATGA CCAAAATCCC TTAACGTGA

//
