# Supplementary material for: Voltage Imaging in Drosophila Using a Hybrid Chemical-Genetic Rhodamine Voltage Reporter
Source: Front Neurosci. 2021 Nov 16;15:754027. doi: 10.3389/fnins.2021.754027 (PMC8637050; doi:10.3389/fnins.2021.754027)
Supplement: Supplementary file 2 [file Table_2.DOCX]

LOCUS pJFRC19-PAT3-Halo(V2)-C 9718 bp ds-DNA circular 03-JUN-2021

DEFINITION .

FEATURES Location/Qualifiers

primer 92..118

/label="pJFRC7 5' Seq 55"

/note="sequence: TGGTGGGCATAATAGTGTTGTTTATAT"

/ApEinfo_revcolor=#faac61

/ApEinfo_fwdcolor=#faac61

primer 429..443

/label="PLEHA 55 5' Bridge Fin"

/note="sequence: CGCAAGCTTGCATGC"

/ApEinfo_revcolor=#c7b0e3

/ApEinfo_fwdcolor=#c7b0e3

misc_feature 432..824

/label="LexAop "

/ApEinfo_revcolor=#85dae9

/ApEinfo_fwdcolor=#85dae9

primer 829..850

/label="Hsp70 promoter seq"

/note="sequence: GAGCGCCGGAGTATAAATAGAG"

/ApEinfo_revcolor=#75c6a9

/ApEinfo_fwdcolor=#75c6a9

misc_feature 832..1059

/label="HS promoter"

/ApEinfo_revcolor=#faac61

/ApEinfo_fwdcolor=#faac61

primer 838..853

/label="20xUASF1"

/note="sequence: AGTATAAATAGAGGCG"

/ApEinfo_revcolor=#75c6a9

/ApEinfo_fwdcolor=#75c6a9

primer complement(954..968)

/label="20xUASR2"

/note="sequence: CACTTTACTGCAGAT"

/ApEinfo_revcolor=#d59687

/ApEinfo_fwdcolor=#d59687

misc_feature 1170..1257

/label="Pat-3 "

/ApEinfo_revcolor=#9eafd2

/ApEinfo_fwdcolor=#9eafd2

CDS 1170..1262

/label="Translation 1170-1262"

misc_feature 1263..2150

/label="HaloTag"

/ApEinfo_revcolor=#c7b0e3

/ApEinfo_fwdcolor=#c7b0e3

CDS 1263..2150

/label="Translation 1263-2150"

primer complement(1323..1341)

/label="Halo Genotyping R2"

/note="sequence: CAACATCGACGTAGTGCAT"

/ApEinfo_revcolor=#faac61

/ApEinfo_fwdcolor=#faac61

primer 1507..1521

/label="Halo Mid Seq F2"

/note="sequence: ACCACGTCCGCTTCA"

/ApEinfo_revcolor=#faac61

/ApEinfo_fwdcolor=#faac61

misc_feature 2151..2180

/label="Linker"

/ApEinfo_revcolor=#85dae9

/ApEinfo_fwdcolor=#85dae9

CDS 2151..2861

/label="Translation 2151-2861"

primer complement(2170..2195)

/label="Halo CD4 R2 NoTag"

/note="sequence: CTTCTGGAAGTCGACCGAGCCTCCAC"

/ApEinfo_revcolor=#c6c9d1

/ApEinfo_fwdcolor=#c6c9d1

misc_feature 2187..2861

/label="CD4 "

/ApEinfo_revcolor=#ff9ccd

/ApEinfo_fwdcolor=#ff9ccd

primer 2387..2404

/label="CD45'seq"

/note="sequence: TAAGCTCCAGATGGGCAA"

/ApEinfo_revcolor=#d6b295

/ApEinfo_fwdcolor=#d6b295

misc_feature 2863..3370

/label="WPRE Ect. "

/ApEinfo_revcolor=#faac61

/ApEinfo_fwdcolor=#faac61

primer complement(2868..2889)

/label="CD4 amp OUT"

/note="sequence: TAAGGTTCCTTCACAAAGATCC"

/ApEinfo_revcolor=#c6c9d1

/ApEinfo_fwdcolor=#c6c9d1

primer 3106..3122

/label="pJFRC7 WPRE F1 55"

/note="sequence: TGAGGCTACTGCTGACT"

/ApEinfo_revcolor=#85dae9

/ApEinfo_fwdcolor=#85dae9

polyA_signal complement(3371..3562)

/label="SV40 late polyA"

/ApEinfo_revcolor=#c6c9d1

/ApEinfo_fwdcolor=#c6c9d1

CDS 3996..4655

/label="AmpR"

/ApEinfo_revcolor=#b7e6d7

/ApEinfo_fwdcolor=#b7e6d7

primer 5211..5230

/label="AttB Sequencing F1V2"

/note="sequence: CAGCGTGAGCTATGAGAAAG"

/ApEinfo_revcolor=#ffef86

/ApEinfo_fwdcolor=#ffef86

misc_feature 5483..5767

/label="AttB"

/ApEinfo_revcolor=#ff9ccd

/ApEinfo_fwdcolor=#ff9ccd

primer 5986..6003

/label="Mini white F1"

/note="sequence: TTGCCTCCTTCTCTGTCC"

/ApEinfo_revcolor=#faac61

/ApEinfo_fwdcolor=#faac61

primer complement(6084..6100)

/label="AttB Sequencing R1V1"

/note="sequence: AATGCAACTGAAGGCGG"

/ApEinfo_revcolor=#b4abac

/ApEinfo_fwdcolor=#b4abac

misc_feature 6617..9271

/label="mini white NGS"

/ApEinfo_revcolor=#f58a5e

/ApEinfo_fwdcolor=#f58a5e

primer 6733..6749

/label="Mini white F2"

/note="sequence: CATTGCAGGGTGACAGC"

/ApEinfo_revcolor=#c6c9d1

/ApEinfo_fwdcolor=#c6c9d1

primer 7451..7468

/label="Mini white F3"

/note="sequence: AGGGTGAAAGGTCTGTCC"

/ApEinfo_revcolor=#faac61

/ApEinfo_fwdcolor=#faac61

primer 8199..8212

/label="Mini white F4"

/note="sequence: GCCTGATTCCACAC"

/ApEinfo_revcolor=#9eafd2

/ApEinfo_fwdcolor=#9eafd2

primer 8199..8214

/label="Mini white F4"

/note="sequence: GCCTGATTCCACACCC"

/ApEinfo_revcolor=#ffef86

/ApEinfo_fwdcolor=#ffef86

primer 8892..8907

/label="Mini white F5"

/note="sequence: TACCTCTCATGGTTCC"

/ApEinfo_revcolor=#faac61

/ApEinfo_fwdcolor=#faac61

ORIGIN

1 TTTTGTGACC TGTTCGGAGT GATTAGCGTT ACAATTTGAA CTGAAAGTGA CATCCAGTGT

61 TTGTTCCTTG TGTAGATGCA TCTCAAAAAA ATGGTGGGCA TAATAGTGTT GTTTATATAT

121 ATCAAAAATA ACAACTATAA TAATAAGAAT ACATTTAATT TAGAAAATGC TTGGATTTCA

181 CTGGAACTAG GGCGCGCCTC CGGAACATAA TGGTGCAGGG CGCTGACTTC CGCGTTTCCA

241 GACTTTACGA AACACGGAAA CCGAAGACCA TTCATGTTGT TGCTCAGGTC GCAGACGTTT

301 TGCAGCAGCA GTCGCTTCAC GTTCGCTCGC GTATCGGTGA TTCATTCTGC TAACCAGTAA

361 GGCAACCCCG CCAGCCTAGC CGGGTCCTCA ACGACAGGAG CACGATCATG CGCACCCGTG

421 GCCAGGGCCG CAAGCTTGCA TGCCTGCAGG TTACTGTACA TCCATACAGT AAGTACTGTA

481 CATCCATACA GTAAGTACTG TACATCCATA CAGTAAGTAC TGTACATCCA TACAGTAAGT

541 ACTGTACATC CATACAGTAA GCGGAGACTC TAGCCCTAGG GCATGCCTGC AGGTTACTGT

601 ACATCCATAC AGTAAGTACT GTACATCCAT ACAGTAAGTA CTGTACATCC ATACAGTAAG

661 CGGAGACTCT AGCGCTAGCG CATGCCTGCA GGTTACTGTA CATCCATACA GTAAGTACTG

721 TACATCCATA CAGTAAGTAC TGTACATCCA TACAGTAAGT ACTGTACATC CATACAGTAA

781 GTACTGTACA TCCATACAGT AAGCGGAGAC TCTAGCACTA GTGACGTCGA GCGCCGGAGT

841 ATAAATAGAG GCGCTTCGTC TACGGAGCGA CAATTCAATT CAAACAAGCA AAGTGAACAC

901 GTCGCTAAGC GAAAGCTAAG CAAATAAACA AGCGCAGCTG AACAAGCTAA ACAATCTGCA

961 GTAAAGTGCA AGTTAAAGTG AATCAATTAA AAGTAACCAG CAACCAAGTA AATCAACTGC

1021 AACTACTGAA ATCTGCCAAG AAGTAATTAT TGAATACAAG AAGAGAACTC TGAATAGATC

1081 TAAAAGGTAG GTTCAACCAC TGATGCCTAG GCACACCGAA ACGACTAACC CTAATTCTTA

1141 TCCTTTACTT CAGGCGGCCG CGGCTCGAGA TGCCACCTTC AACATCATTG CTGCTCCTCG

1201 CAGCACTTCT TCCATTCGCT TTACCAGCAA GCGATTGGAA GACTGGAGAA GTCACTGCTA

1261 GCGCAGAAAT CGGTACTGGC TTTCCATTCG ACCCCCATTA TGTGGAAGTC CTGGGCGAGC

1321 GCATGCACTA CGTCGATGTT GGTCCGCGCG ATGGCACCCC TGTGCTGTTC CTGCACGGTA

1381 ACCCGACCTC CTCCTACGTG TGGCGCAACA TCATCCCGCA TGTTGCACCG ACCCATCGCT

1441 GCATTGCTCC AGACCTGATC GGTATGGGCA AATCCGACAA ACCAGACCTG GGTTATTTCT

1501 TCGACGACCA CGTCCGCTTC ATGGATGCCT TCATCGAAGC CCTGGGTCTG GAAGAGGTCG

1561 TCCTGGTCAT TCACGACTGG GGCTCCGCTC TGGGTTTCCA CTGGGCCAAG CGCAATCCAG

1621 AGCGCGTCAA AGGTATTGCA TTTATGGAGT TCATCCGCCC TATCCCGACC TGGGACGAAT

1681 GGCCAGAATT TGCCCGCGAG ACCTTCCAGG CCTTCCGCAC CACCGACGTC GGCCGCAAGC

1741 TGATCATCGA TCAGAACGTT TTTATCGAGG GTACGCTGCC GATGGGTGTC GTCCGCCCGC

1801 TGACTGAAGT CGAGATGGAC CATTACCGCG AGCCGTTCCT GAATCCTGTT GACCGCGAGC

1861 CACTGTGGCG CTTCCCAAAC GAGCTGCCAA TCGCCGGTGA GCCAGCGAAC ATCGTCGCGC

1921 TGGTCGAAGA ATACATGGAC TGGCTGCACC AGTCCCCTGT CCCGAAGCTG CTGTTCTGGG

1981 GCACCCCAGG CGTTCTGATC CCACCGGCCG AAGCCGCTCG CCTGGCCAAA AGCCTGCCTA

2041 ACTGCAAGGC TGTGGACATC GGCCCGGGTC TGAATCTGCT GCAAGAAGAC AACCCGGACC

2101 TGATCGGCAG CGAGATCGCG CGCTGGCTGT CGACGCTCGA GATTTCCGGC GGTGGCGGCG

2161 GAAGTGGAGG TGGAGGCTCG GTCGACTTCC AGAAGGCCTC CAGCATAGTC TATAAGAAAG

2221 AGGGGGAACA GGTGGAGTTC TCCTTCCCAC TCGCCTTTAC AGTTGAAAAG CTGACGGGCA

2281 GTGGCGAGCT GTGGTGGCAG GCGGAGAGGG CTTCCTCCTC CAAGTCTTGG ATCACCTTTG

2341 ACCTGAAGAA CAAGGAAGTG TCTGTAAAAC GGGTTACCCA GGACCCTAAG CTCCAGATGG

2401 GCAAGAAGCT CCCGCTCCAC CTCACCCTGC CCCAGGCCTT GCCTCAGTAT GCTGGCTCTG

2461 GAAACCTCAC CCTGGCCCTT GAAGCGAAAA CAGGAAAGTT GCATCAGGAA GTGAACCTGG

2521 TGGTGATGAG AGCCACTCAG CTCCAGAAAA ATTTGACCTG TGAGGTGTGG GGACCCACCT

2581 CCCCTAAGCT GATGCTGAGC TTGAAACTGG AGAACAAGGA GGCAAAGGTC TCGAAGCGGG

2641 AGAAGGCGGT GTGGGTGCTG AACCCTGAGG CGGGGATGTG GCAGTGTCTG CTGAGTGACT

2701 CGGGACAGGT CCTGCTGGAA TCCAACATCA AGGTTCTGCC CACATGGTCC ACCCCGGTGC

2761 AGCCAATGGC CCTGATTGTG CTGGGGGGCG TCGCCGGCCT CCTGCTTTTC ATTGGGCTAG

2821 GCATCTTCTT CTGTGTCAGG TGCCGGCACC GAAGGCGCTA GTCTAGAGGA TCTTTGTGAA

2881 GGAACCTTAC TTCTGTGGTG TGACATAATT GGACAAACTA CCTACAGAGA TTTAAAGCTC

2941 TAAGGTAAAT ATAAAATTTT TAAGTGTATA ATGTGTTAAA CTACTGATTC TAATTGTTTG

3001 TGTATTTTAG ATTCCAACCT ATGGAACTGA TGAATGGGAG CAGTGGTGGA ATGCCTTTAA

3061 TGAGGAAAAC CTGTTTTGCT CAGAAGAAAT GCCATCTAGT GATGATGAGG CTACTGCTGA

3121 CTCTCAACAT TCTACTCCTC CAAAAAAGAA GAGAAAGGTA GAAGACCCCA AGGACTTTCC

3181 TTCAGAATTG CTAAGTTTTT TGAGTCATGC TGTGTTTAGT AATAGAACTC TTGCTTGCTT

3241 TGCTATTTAC ACCACAAAGG AAAAAGCTGC ACTGCTATAC AAGAAAATTA TGGAAAAATA

3301 TTTGATGTAT AGTGCCTTGA CTAGAGATCA TAATCAGCCA TACCACATTT GTAGAGGTTT

3361 TACTTGCTTT AAAAAACCTC CCACACCTCC CCCTGAACCT GAAACATAAA ATGAATGCAA

3421 TTGTTGTTGT TAACTTGTTT ATTGCAGCTT ATAATGGTTA CAAATAAAGC AATAGCATCA

3481 CAAATTTCAC AAATAAAGCA TTTTTTTCAC TGCATTCTAG TTGTGGTTTG TCCAAACTCA

3541 TCAATGTATC TTATCATGTC TGGATCGATC TGGCCGGCCG TTTAAACGAA TTCTTGAAGA

3601 CGAAAGGGCC TCGTGATACG CCTATTTTTA TAGGTTAATG TCATGATAAT AATGGTTTCT

3661 TAGACTCAGG TGGCACTTTT CGGGGAAATG TGCGCGGAAC CCCTATTTGT TTATTTTTCT

3721 AAATACATTC AAATATGTAT CCGCTCATGA GACAATAACC CTGATAAATG CTTCAATAAT

3781 ATTGAAAAAG GAAGAGTATG AGTATTCAAC ATTTCCGTGT CGCCCTTATT CCCTTTTTTG

3841 CGGCATTTTG CCTTCCTGTT TTTGCTCACC CAGAAACGCT GGTGAAAGTA AAAGATGCTG

3901 AAGATCAGTT GGGTGCACGA GTGGGTTACA TCGAACTGGA TCTCAACAGC GGTAAGATCC

3961 TTGAGAGTTT TCGCCCCGAA GAACGTTTTC CAATGATGAG CACTTTTAAA GTTCTGCTAT

4021 GTGGCGCGGT ATTATCCCGT ATTGACGCCG GGCAAGAGCA ACTCGGTCGC CGCATACACT

4081 ATTCTCAGAA TGACTTGGTT GAGTACTCAC CAGTCACAGA AAAGCATCTT ACGGATGGCA

4141 TGACAGTAAG AGAATTATGC AGTGCTGCCA TAACCATGAG TGATAACACT GCGGCCAACT

4201 TACTTCTGAC AACGATCGGA GGACCGAAGG AGCTAACCGC TTTTTTGCAC AACATGGGGG

4261 ATCATGTAAC TCGCCTTGAT CGTTGGGAAC CGGAGCTGAA TGAAGCCATA CCAAACGACG

4321 AGCGTGACAC CACGATGCCT GTAGCAATGG CAACAACGTT GCGCAAACTA TTAACTGGCG

4381 AACTACTTAC TCTAGCTTCC CGGCAACAAT TAATAGACTG GATGGAGGCG GATAAAGTTG

4441 CAGGACCACT TCTGCGCTCG GCCCTTCCGG CTGGCTGGTT TATTGCTGAT AAATCTGGAG

4501 CCGGTGAGCG TGGGTCTCGC GGTATCATTG CAGCACTGGG GCCAGATGGT AAGCCCTCCC

4561 GTATCGTAGT TATCTACACG ACGGGGAGTC AGGCAACTAT GGATGAACGA AATAGACAGA

4621 TCGCTGAGAT AGGTGCCTCA CTGATTAAGC ATTGGTAACT GTCAGACCAA GTTTACTCAT

4681 ATATACTTTA GATTGATTTA AAACTTCATT TTTAATTTAA AAGGATCTAG GTGAAGATCC

4741 TTTTTGATAA TCTCATGACC AAAATCCCTT AACGTGAGTT TTCGTTCCAC TGAGCGTCAG

4801 ACCCCGTAGA AAAGATCAAA GGATCTTCTT GAGATCCTTT TTTTCTGCGC GTAATCTGCT

4861 GCTTGCAAAC AAAAAAACCA CCGCTACCAG CGGTGGTTTG TTTGCCGGAT CAAGAGCTAC

4921 CAACTCTTTT TCCGAAGGTA ACTGGCTTCA GCAGAGCGCA GATACCAAAT ACTGTTCTTC

4981 TAGTGTAGCC GTAGTTAGGC CACCACTTCA AGAACTCTGT AGCACCGCCT ACATACCTCG

5041 CTCTGCTAAT CCTGTTACCA GTGGCTGCTG CCAGTGGCGA TAAGTCGTGT CTTACCGGGT

5101 TGGACTCAAG ACGATAGTTA CCGGATAAGG CGCAGCGGTC GGGCTGAACG GGGGGTTCGT

5161 GCACACAGCC CAGCTTGGAG CGAACGACCT ACACCGAACT GAGATACCTA CAGCGTGAGC

5221 TATGAGAAAG CGCCACGCTT CCCGAAGGGA GAAAGGCGGA CAGGTATCCG GTAAGCGGCA

5281 GGGTCGGAAC AGGAGAGCGC ACGAGGGAGC TTCCAGGGGG AAACGCCTGG TATCTTTATA

5341 GTCCTGTCGG GTTTCGCCAC CTCTGACTTG AGCGTCGATT TTTGTGATGC TCGTCAGGGG

5401 GGCGGAGCCT ATGGAAAAAC GCCAGCAACG CGGCCTTTTT ACGGTTCCTG GCCTTTTGCT

5461 GGCCTTTTGC TCACATGTTA CCGTCGACGA TGTAGGTCAC GGTCTCGAAG CCGCGGTGCG

5521 GGTGCCAGGG CGTGCCCTTG GGCTCCCCGG GCGCGTACTC CACCTCACCC ATCTGGTCCA

5581 TCATGATGAA CGGGTCGAGG TGGCGGTAGT TGATCCCGGC GAACGCGCGG CGCACCGGGA

5641 AGCCCTCGCC CTCGAAACCG CTGGGCGCGG TGGTCACGGT GAGCACGGGA CGTGCGACGG

5701 CGTCGGCGGG TGCGGATACG CGGGGCAGCG TCAGCGGGTT CTCGACGGTC ACGGCGGGCA

5761 TGTCGACAAG CCGAACATAT GGGCGCGCCT AGTATGTATG TAAGTTAATA AAACCCATTT

5821 TTGCGGAAAG TAGATAAAAA AAACATTTTT TTTTTTTACT GCACTGGATA TCATTGAACT

5881 TATCTGATCA GTTTTAAATT TACTTCGATC CAAGGGTATT TGATGTACCA GGTTCTTTCG

5941 ATTACCTCTC ACTCAAAATG ACATTCCACT CAAAGTCAGC GCTGTTTGCC TCCTTCTCTG

6001 TCCACAGAAA TATCGCCGTC TCTTTCGCCG CTGCGTCCGC TATCTCTTTC GCCACCGTTT

6061 GTAGCGTTAC GTAGCGTCAA TGTCCGCCTT CAGTTGCATT TTGTCAGCGG TTTCGTGACG

6121 AAGCTCCAAG CGGTTTACGC CATCAATTAA ACACAAAGTG CTGTGCCAAA ACTCCTCTCG

6181 CTTCTTATTT TTGTTTGTTT TTTGAGTGAT TGGGGTGGTG ATTGGTTTTG GGTGGGTAAG

6241 CAGGGGAAAG TGTGAAAAAT CCCGGCAATG GGCCAAGAGG ATCAGGAGCT ATTAATTCGC

6301 GGAGGCAGCA AACACCCATC TGCCGAGCAT CTGAACAATG TGAGTAGTAC ATGTGCATAC

6361 ATCTTAAGTT CACTTGATCT ATAGGAACTG CGATTGCAAC ATCAAATTGT CTGCGGCGTG

6421 AGAACTGCGA CCCACAAAAA TCCCAAACCG CAATTGCACA AACAAATAGT GACACGAAAC

6481 AGATTATTCT GGTAGCTGTT CTCGCTATAT AAGACAATTT TTGAGATCAT ATCATGATCA

6541 AGACATCTAA AGGCATTCAT TTTCGACTAT ATTCTTTTTT ACAAAAAATA TAACAACCAG

6601 ATATTTTAAG CTGATCCTAG ATGCACAAAA AATAAATAAA AGTATAAACC TACTTCGTAG

6661 GATACTTCGG GGTACTTTTT GTTCGGGGTT AGATGAGCAT AACGCTTGTA GTTGATATTT

6721 GAGATCCCCT ATCATTGCAG GGTGACAGCG GAGCGGCTTC GCAGAGCTGC ATTAACCAGG

6781 GCTTCGGGCA GGCCAAAAAC TACGGCACGC TCCGGCCACC CAGTCCGCCG GAGGACTCCG

6841 GTTCAGGGAG CGGCCAACTA GCCGAGAACC TCACCTATGC CTGGCACAAT ATGGACATCT

6901 TTGGGGCGGT CAATCAGCCG GGCTCCGGAT GGCGGCAGCT GGTCAACCGG ACACGCGGAC

6961 TATTCTGCAA CGAGCGACAC ATACCGGCGC CCAGGAAACA TTTGCTCAAG AACGGTGAGT

7021 TTCTATTCGC AGTCGGCTGA TCTGTGTGAA ATCTTAATAA AGGGTCCAAT TACCAATTTG

7081 AAACTCAGTT TGCGGCGTGG CCTATCCGGG CGAACTTTTG GCCGTGATGG GCAGTTCCGG

7141 TGCCGGAAAG ACGACCCTGC TGAATGCCCT TGCCTTTCGA TCGCCGCAGG GCATCCAAGT

7201 ATCGCCATCC GGGATGCGAC TGCTCAATGG CCAACCTGTG GACGCCAAGG AGATGCAGGC

7261 CAGGTGCGCC TATGTCCAGC AGGATGACCT CTTTATCGGC TCCCTAACGG CCAGGGAACA

7321 CCTGATTTTC CAAGCCATGG TGCGGATGCC ACGACATCTG ACCTATCGGC AGCGAGTGGC

7381 CCGCGTGGAT CAGGTGATCC AGGAGCTTTC GCTCAGCAAA TGTCAGCACA CGATCATCGG

7441 TGTGCCCGGC AGGGTGAAAG GTCTGTCCGG CGGAGAAAGG AAGCGTCTGG CATTCGCCTC

7501 CGAGGCTCTA ACCGATCCGC CGCTTCTGAT CTGCGATGAG CCCACCTCCG GACTGGACTC

7561 CTTTACCGCC CACAGCGTCG TCCAGGTGCT GAAGAAGCTG TCGCAGAAGG GCAAGACCGT

7621 CATCCTGACC ATTCATCAGC CGTCTTCCGA GCTGTTTGAG CTCTTTGACA AGATCCTTCT

7681 GATGGCCGAG GGCAGGGTAG CTTTCTTGGG CACTCCCAGC GAAGCCGTCG ACTTCTTTTC

7741 CTAGTGAGTT CGATGTGTTT ATTAAGGGTA TCTAGTATTA CATAACATCT CAACTCCTAT

7801 CCAGCGTGGG TGCCCAGTGT CCTACCAACT ACAATCCGGC GGACTTTTAC GTACAGGTGT

7861 TGGCCGTTGT GCCCGGACGG GAGATCGAGT CCCGTGATCG GATCGCCAAG ATATGCGACA

7921 ATTTTGCCAT TAGCAAAGTA GCCCGGGATA TGGAGCAGTT GTTGGCCACC AAAAATCTGG

7981 AGAAGCCACT GGAGCAGCCG GAGAATGGGT ACACCTACAA GGCCACCTGG TTCATGCAGT

8041 TCCGGGCGGT CCTGTGGCGA TCCTGGCTGT CGGTGCTCAA GGAACCACTC CTCGTAAAAG

8101 TGCGACTTAT TCAGACAACG GTGAGTGGTT CCAGTGGAAA CAAATGATAT AACGCTTACA

8161 ATTCTTGGAA ACAAATTCGC TAGATTTTAG ATAGAATTGC CTGATTCCAC ACCCTTCTTA

8221 GTTTTTTTCA ATGAGATGTA TAGTTTATAG TTTTGCAGAA GATAAATAAA TTTCATTTAA

8281 CTCGCGAATA TTAATGAGAT GCGAGTAACA TTTTAATTTG CAGATGGTTG CCATCTTGAT

8341 TGGCCTCATC TTTTTGGGCC AACAACTCAC GCAAGTGGGT GTGATGAATA TCAACGGAGC

8401 CATCTTCCTC TTCCTGACCA ACATGACCTT TCAAAACGTC TTTGCCACGA TAAATGTAAG

8461 TCATGTTTAG AATACATTTG CATTTCAATA ATTTACTAAC TTTCTAATGA ATCGATTCGA

8521 TTTAGGTGTT CACCTCAGAG CTGCCAGTTT TTATGAGGGA GGCCCGAAGT CGACTTTATC

8581 GCTGTGACAC ATACTTTCTG GGCAAAACGA TTGCCGAATT GCCGCTTTTT CTCACAGTGC

8641 CACTGGTCTT CACGGCGATT GCCTATCCGA TGATCGGACT GCGGGCCGGA GTGCTGCACT

8701 TCTTCAACTG CCTGGCGCTG GTCACTCTGG TGGCCAATGT GTCAACGTCC TTCGGATATC

8761 TAATATCCTG CGCCAGCTCC TCGACCTCGA TGGCGCTGTC TGTGGGTCCG CCGGTTATCA

8821 TACCATTCCT GCTCTTTGGC GGCTTCTTCT TGAACTCGGG CTCGGTGCCA GTATACCTCA

8881 AATGGTTGTC GTACCTCTCA TGGTTCCGTT ACGCCAACGA GGGTCTGCTG ATTAACCAAT

8941 GGGCGGACGT GGAGCCGGGC GAAATTAGCT GCACATCGTC GAACACCACG TGCCCCAGTT

9001 CGGGCAAGGT CATCCTGGAG ACGCTTAACT TCTCCGCCGC CGATCTGCCG CTGGACTACG

9061 TGGGTCTGGC CATTCTCATC GTGAGCTTCC GGGTGCTCGC ATATCTGGCT CTAAGACTTC

9121 GGGCCCGACG CAAGGAGTAG CCGACATATA TCCGAAATAA CTGCTTGTTT TTTTTTTTTA

9181 CCATTATTAC CATCGTGTTT ACTGTTTATT GCCCCCTCAA AAAGCTAATG TAATTATATT

9241 TGTGCCAATA AAAACAAGAT ATGACCTATA GAATACAAGT ATTTCCCCTT CGAACATCCC

9301 CACAAGTAGA CTTTGGATTT GTCTTCTAAC CAAAAGACTT ACACACCTGC ATACCTTACA

9361 TCAAAAACTC GTTTATCGCT ACATAAAACA CCGGGATATA TTTTTTATAT ACATACTTTT

9421 CAAATCGCGC GCCCTCTTCA TAATTCACCT CCACCACACC ACGTTTCGTA GTTGCTCTTT

9481 CGCTGTCTCC CACCCGCTCT CCGCAACACA TTCACCTTTT GTTCGACGAC CTTGGAGCGA

9541 CTGTCGTTAG TTCCGCGCGA TTCGGTTCGC TCAAATGGTT CCGAGTGGTT CATTTCGTCT

9601 CAATAGAAAT TAGTAATAAA TATTTGTATG TACAATTTAT TTGCTCCAAT ATATTTGTAT

9661 ATATTTCCCT CACAGCTATA TTTATTCTAA TTTAATATTA TGACTTTTTA AGGTAATT

//
