# Supplementary material for: Voltage Imaging in Drosophila Using a Hybrid Chemical-Genetic Rhodamine Voltage Reporter
Source: Front Neurosci. 2021 Nov 16;15:754027. doi: 10.3389/fnins.2021.754027 (PMC8637050; doi:10.3389/fnins.2021.754027)
Supplement: Supplementary file 3 [file Table_3.DOCX]

LOCUS pcDNA3_Halo_(V2)_CD4_2_ 6679 bp ds-DNA circular 03-JUN-2021

DEFINITION .

KEYWORDS "creator:Trial User" "marker:AmpR"

FEATURES Location/Qualifiers

promoter complement(428..788)

/label="SV40 promoter"

/ApEinfo_revcolor=#c6c9d1

/ApEinfo_fwdcolor=#c6c9d1

CDS complement(848..1639)

/label="AmpR"

/ApEinfo_revcolor=#b7e6d7

/ApEinfo_fwdcolor=#b7e6d7

promoter complement(1709..1813)

/label="AmpR promoter"

/ApEinfo_revcolor=#c6c9d1

/ApEinfo_fwdcolor=#c6c9d1

enhancer 2307..2686

/label="CMV enhancer"

/ApEinfo_revcolor=#85dae9

/ApEinfo_fwdcolor=#85dae9

promoter 2687..2890

/label="CMV promoter"

/ApEinfo_revcolor=#f58a5e

/ApEinfo_fwdcolor=#f58a5e

promoter 2935..2953

/label="T7 promoter"

/ApEinfo_revcolor=#b1ff67

/ApEinfo_fwdcolor=#b1ff67

primer 3016..3033

/label="Pat3 Fwd"

/note="sequence: ATGCCACCTTCAACATCA"

/ApEinfo_revcolor=#b4abac

/ApEinfo_fwdcolor=#b4abac

misc_feature 3016..3103

/label="Pat-3 "

/ApEinfo_revcolor=#9eafd2

/ApEinfo_fwdcolor=#9eafd2

CDS 3016..3103

/label="Translation 3016-3103"

CDS 3104..3108

/label="Translation 3104-3108"

misc_feature 3109..3996

/label="HaloTag"

/ApEinfo_revcolor=#c7b0e3

/ApEinfo_fwdcolor=#c7b0e3

CDS 3109..3996

/label="Translation 3109-3996"

CDS 3109..3996

/label="Translation 3109-3996"

CDS 3109..3996

/label="Translation 3109-3996"

misc_feature 3997..4026

/label="Linker"

/ApEinfo_revcolor=#85dae9

/ApEinfo_fwdcolor=#85dae9

CDS 3997..4026

/label="Translation 3997-4026"

CDS 4027..4707

/label="Translation 4027-4707"

misc_feature 4033..4707

/label="CD4 "

/ApEinfo_revcolor=#ff9ccd

/ApEinfo_fwdcolor=#ff9ccd

CDS 4132..4151

/label="Translation 4132-4151"

CDS 4708..4956

/label="Translation 4708-4956"

polyA_signal 4732..4956

/label="bGH poly(A) signal"

/ApEinfo_revcolor=#b4abac

/ApEinfo_fwdcolor=#b4abac

ORIGIN

1 GCAGGTAGCC GGATCAAGCG TATGCAGCCG CCGCATTGCA TCAGCCATGA TGGATACTTT

61 CTCGGCAGGA GCAAGGTGAG ATGACAGGAG ATCCTGCCCC GGCACTTCGC CCAATAGCAG

121 CCAGTCCCTT CCCGCTTCAG TGACAACGTC GAGCACAGCT GCGCAAGGAA CGCCCGTCGT

181 GGCCAGCCAC GATAGCCGCG CTGCCTCGTC TTGCAGTTCA TTCAGGGCAC CGGACAGGTC

241 GGTCTTGACA AAAAGAACCG GGCGCCCCTG CGCTGACAGC CGGAACACGG CGGCATCAGA

301 GCAGCCGATT GTCTGTTGTG CCCAGTCATA GCCGAATAGC CTCTCCACCC AAGCGGCCGG

361 AGAACCTGCG TGCAATCCAT CTTGTTCAAT CATGCGAAAC GATCCTCATC CTGTCTCTTG

421 ATCGATCTTT GCAAAAGCCT AGGCCTCCAA AAAAGCCTCC TCACTACTTC TGGAATAGCT

481 CAGAGGCCGA GGAGGCGGCC TCGGCCTCTG CATAAATAAA AAAAATTAGT CAGCCATGGG

541 GCGGAGAATG GGCGGAACTG GGCGGAGTTA GGGGCGGGAT GGGCGGAGTT AGGGGCGGGA

601 CTATGGTTGC TGACTAATTG AGATGCATGC TTTGCATACT TCTGCCTGCT GGGGAGCCTG

661 GGGACTTTCC ACACCTGGTT GCTGACTAAT TGAGATGCAT GCTTTGCATA CTTCTGCCTG

721 CTGGGGAGCC TGGGGACTTT CCACACCCTA ACTGACACAC ATTCCACAGC TGGTTCTTTC

781 CGCCTCAGGA CTCTTCCTTT TTCAATAAAT CAATCTAAAG TATATATGAG TAAACTTGGT

841 CTGACAGTTA CCAATGCTTA ATCAGTGAGG CACCTATCTC AGCGATCTGT CTATTTCGTT

901 CATCCATAGT TGCCTGACTC CCCGTCGTGT AGATAACTAC GATACGGGAG GGCTTACCAT

961 CTGGCCCCAG TGCTGCAATG ATACCGCGAG ACCCACGCTC ACCGGCTCCA GATTTATCAG

1021 CAATAAACCA GCCAGCCGGA AGGGCCGAGC GCAGAAGTGG TCCTGCAACT TTATCCGCCT

1081 CCATCCAGTC TATTAATTGT TGCCGGGAAG CTAGAGTAAG TAGTTCGCCA GTTAATAGTT

1141 TGCGCAACGT TGTTGCCATT GCTACAGGCA TCGTGGTGTC ACGCTCGTCG TTTGGTATGG

1201 CTTCATTCAG CTCCGGTTCC CAACGATCAA GGCGAGTTAC ATGATCCCCC ATGTTGTGCA

1261 AAAAAGCGGT TAGCTCCTTC GGTCCTCCGA TCGTTGTCAG AAGTAAGTTG GCCGCAGTGT

1321 TATCACTCAT GGTTATGGCA GCACTGCATA ATTCTCTTAC TGTCATGCCA TCCGTAAGAT

1381 GCTTTTCTGT GACTGGTGAG TACTCAACCA AGTCATTCTG AGAATAGTGT ATGCGGCGAC

1441 CGAGTTGCTC TTGCCCGGCG TCAATACGGG ATAATACCGC GCCACATAGC AGAACTTTAA

1501 AAGTGCTCAT CATTGGAAAA CGTTCTTCGG GGCGAAAACT CTCAAGGATC TTACCGCTGT

1561 TGAGATCCAG TTCGATGTAA CCCACTCGTG CACCCAACTG ATCTTCAGCA TCTTTTACTT

1621 TCACCAGCGT TTCTGGGTGA GCAAAAACAG GAAGGCAAAA TGCCGCAAAA AAGGGAATAA

1681 GGGCGACACG GAAATGTTGA ATACTCATAC TCTTCCTTTT TCAATATTAT TGAAGCATTT

1741 ATCAGGGTTA TTGTCTCATG AGCGGATACA TATTTGAATG TATTTAGAAA AATAAACAAA

1801 TAGGGGTTCC GCGCACATTT CCCCGAAAAG TGCCACCTGA CGCGCCCTGT AGCGGCGCAT

1861 TAAGCGCGGC GGGTGTGGTG GTTACGCGCA GCGTGACCGC TACACTTGCC AGCGCCCTAG

1921 CGCCCGCTCC TTTCGCTTTC TTCCCTTCCT TTCTCGCCAC GTTCGCCGGC TTTCCCCGTC

1981 AAGCTCTAAA TCGGGGGCTC CCTTTAGGGT TCCGATTTAG TGCTTTACGG CACCTCGACC

2041 CCAAAAAACT TGATTAGGGT GATGGTTCAC GTAGTGGGCC ATCGCCCTGA TAGACGGTTT

2101 TTCGCCCTTT GACGTTGGAG TCCACGTTCT TTAATAGTGG ACTCTTGTTC CAAACTGGAA

2161 CAACACTCAA CCCTATCTCG GTCTATTCTT TTGATTTATA AGGGATTTTG CCGATTTCGG

2221 CCTATTGGTT AAAAAATGAG CTGATTTAAC AAAAATTTAA CGCGAATTTT AACAAAATAT

2281 TAACGCTTAC AATTTACGCG CGCGTTGACA TTGATTATTG ACTAGTTATT AATAGTAATC

2341 AATTACGGGG TCATTAGTTC ATAGCCCATA TATGGAGTTC CGCGTTACAT AACTTACGGT

2401 AAATGGCCCG CCTGGCTGAC CGCCCAACGA CCCCCGCCCA TTGACGTCAA TAATGACGTA

2461 TGTTCCCATA GTAACGCCAA TAGGGACTTT CCATTGACGT CAATGGGTGG ACTATTTACG

2521 GTAAACTGCC CACTTGGCAG TACATCAAGT GTATCATATG CCAAGTACGC CCCCTATTGA

2581 CGTCAATGAC GGTAAATGGC CCGCCTGGCA TTATGCCCAG TACATGACCT TATGGGACTT

2641 TCCTACTTGG CAGTACATCT ACGTATTAGT CATCGCTATT ACCATGGTGA TGCGGTTTTG

2701 GCAGTACATC AATGGGCGTG GATAGCGGTT TGACTCACGG GGATTTCCAA GTCTCCACCC

2761 CATTGACGTC AATGGGAGTT TGTTTTGGCA CCAAAATCAA CGGGACTTTC CAAAATGTCG

2821 TAACAACTCC GCCCCATTGA CGCAAATGGG CGGTAGGCGT GTACGGTGGG AGGTCTATAT

2881 AAGCAGAGCT CTCTGGCTAA CTAGAGAACC CACTGCTTAC TGGCTTATCG AAATTAATAC

2941 GACTCACTAT AGGGAGACCC AAGCTTGGTA CCGAGCTCGG ATCCACTAGT AACGGCCGCC

3001 AGTGTGCTGG AATTCATGCC ACCTTCAACA TCATTGCTGC TCCTCGCAGC ACTTCTTCCA

3061 TTCGCTTTAC CAGCAAGCGA TTGGAAGACT GGAGAAGTCA CTGCTAGCGC AGAAATCGGT

3121 ACTGGCTTTC CATTCGACCC CCATTATGTG GAAGTCCTGG GCGAGCGCAT GCACTACGTC

3181 GATGTTGGTC CGCGCGATGG CACCCCTGTG CTGTTCCTGC ACGGTAACCC GACCTCCTCC

3241 TACGTGTGGC GCAACATCAT CCCGCATGTT GCACCGACCC ATCGCTGCAT TGCTCCAGAC

3301 CTGATCGGTA TGGGCAAATC CGACAAACCA GACCTGGGTT ATTTCTTCGA CGACCACGTC

3361 CGCTTCATGG ATGCCTTCAT CGAAGCCCTG GGTCTGGAAG AGGTCGTCCT GGTCATTCAC

3421 GACTGGGGCT CCGCTCTGGG TTTCCACTGG GCCAAGCGCA ATCCAGAGCG CGTCAAAGGT

3481 ATTGCATTTA TGGAGTTCAT CCGCCCTATC CCGACCTGGG ACGAATGGCC AGAATTTGCC

3541 CGCGAGACCT TCCAGGCCTT CCGCACCACC GACGTCGGCC GCAAGCTGAT CATCGATCAG

3601 AACGTTTTTA TCGAGGGTAC GCTGCCGATG GGTGTCGTCC GCCCGCTGAC TGAAGTCGAG

3661 ATGGACCATT ACCGCGAGCC GTTCCTGAAT CCTGTTGACC GCGAGCCACT GTGGCGCTTC

3721 CCAAACGAGC TGCCAATCGC CGGTGAGCCA GCGAACATCG TCGCGCTGGT CGAAGAATAC

3781 ATGGACTGGC TGCACCAGTC CCCTGTCCCG AAGCTGCTGT TCTGGGGCAC CCCAGGCGTT

3841 CTGATCCCAC CGGCCGAAGC CGCTCGCCTG GCCAAAAGCC TGCCTAACTG CAAGGCTGTG

3901 GACATCGGCC CGGGTCTGAA TCTGCTGCAA GAAGACAACC CGGACCTGAT CGGCAGCGAG

3961 ATCGCGCGCT GGCTGTCGAC GCTCGAGATT TCCGGCGGTG GCGGCGGAAG TGGAGGTGGA

4021 GGCTCGGTCG ACTTCCAGAA GGCCTCCAGC ATAGTCTATA AGAAAGAGGG GGAACAGGTG

4081 GAGTTCTCCT TCCCACTCGC CTTTACAGTT GAAAAGCTGA CGGGCAGTGG CGAGCTGTGG

4141 TGGCAGGCGG AGAGGGCTTC CTCCTCCAAG TCTTGGATCA CCTTTGACCT GAAGAACAAG

4201 GAAGTGTCTG TAAAACGGGT TACCCAGGAC CCTAAGCTCC AGATGGGCAA GAAGCTCCCG

4261 CTCCACCTCA CCCTGCCCCA GGCCTTGCCT CAGTATGCTG GCTCTGGAAA CCTCACCCTG

4321 GCCCTTGAAG CGAAAACAGG AAAGTTGCAT CAGGAAGTGA ACCTGGTGGT GATGAGAGCC

4381 ACTCAGCTCC AGAAAAATTT GACCTGTGAG GTGTGGGGAC CCACCTCCCC TAAGCTGATG

4441 CTGAGCTTGA AACTGGAGAA CAAGGAGGCA AAGGTCTCGA AGCGGGAGAA GGCGGTGTGG

4501 GTGCTGAACC CTGAGGCGGG GATGTGGCAG TGTCTGCTGA GTGACTCGGG ACAGGTCCTG

4561 CTGGAATCCA ACATCAAGGT TCTGCCCACA TGGTCCACCC CGGTGCAGCC AATGGCCCTG

4621 ATTGTGCTGG GGGGCGTCGC CGGCCTCCTG CTTTTCATTG GGCTAGGCAT CTTCTTCTGT

4681 GTCAGGTGCC GGCACCGAAG GCGCTAGGCG GCCGCTCGAG ATCAGCCTCG ACTGTGCCTT

4741 CTAGTTGCCA GCCATCTGTT GTTTGCCCCT CCCCCGTGCC TTCCTTGACC CTGGAAGGTG

4801 CCACTCCCAC TGTCCTTTCC TAATAAAATG AGGAAATTGC ATCGCATTGT CTGAGTAGGT

4861 GTCATTCTAT TCTGGGGGGT GGGGTGGGGC AGGACAGCAA GGGGGAGGAT TGGGAAGACA

4921 ATAGCAGGCA TGCTGGGGAT GCGGTGGGCT CTATGGCTTC TGAGGCGGAA AGAACCAGTG

4981 GCGGTAATAC GGTTATCCAC AGAATCAGGG GATAACGCAG GAAAGAACAT GTGAGCAAAA

5041 GGCCAGCAAA AGGCCAGGAA CCGTAAAAAG GCCGCGTTGC TGGCGTTTTT CCATAGGCTC

5101 CGCCCCCCTG ACGAGCATCA CAAAAATCGA CGCTCAAGTC AGAGGTGGCG AAACCCGACA

5161 GGACTATAAA GATACCAGGC GTTTCCCCCT GGAAGCTCCC TCGTGCGCTC TCCTGTTCCG

5221 ACCCTGCCGC TTACCGGATA CCTGTCCGCC TTTCTCCCTT CGGGAAGCGT GGCGCTTTCT

5281 CATAGCTCAC GCTGTAGGTA TCTCAGTTCG GTGTAGGTCG TTCGCTCCAA GCTGGGCTGT

5341 GTGCACGAAC CCCCCGTTCA GCCCGACCGC TGCGCCTTAT CCGGTAACTA TCGTCTTGAG

5401 TCCAACCCGG TAAGACACGA CTTATCGCCA CTGGCAGCAG CCACTGGTAA CAGGATTAGC

5461 AGAGCGAGGT ATGTAGGCGG TGCTACAGAG TTCTTGAAGT GGTGGCCTAA CTACGGCTAC

5521 ACTAGAAGGA CAGTATTTGG TATCTGCGCT CTGCTGAAGC CAGTTACCTT CGGAAAAAGA

5581 GTTGGTAGCT CTTGATCCGG CAAACAAACC ACCGCTGGTA GCGGTGGTTT TTTTGTTTGC

5641 AAGCAGCAGA TTACGCGCAG AAAAAAAGGA TCTCAAGAAG ATCCTTTGAT CTTTTCTACG

5701 GGGTCTGACG CTCAGTGGAA CGAAAACTCA CGTTAAGGGA TTTTGGTCAT GAGATTATCA

5761 AAAAGGATCT TCACCTAGAT CCTTTTAAAT TAAAAATGAA GTTTTAAATC AATCTAAAGT

5821 ATATATGAGT AACCTGAGGC TATGGCAGGG CCTGCCGCCC CGACGTTGGC TGCGAGCCCT

5881 GGGCCTTCAC CCGAACTTGG GGGGTGGGGT GGGGAAAAGG AAGAAACGCG GGCGTATTGG

5941 CCCCAATGGG GTCTCGGTGG GGTATCGACA GAGTGCCAGC CCTGGGACCG AACCCCGCGT

6001 TTATGAACAA ACGACCCAAC ACCGTGCGTT TTATTCTGTC TTTTTATTGC CGTCATAGCG

6061 CGGGTTCCTT CCGGTATTGT CTCCTTCCGT GTTTCAGTTA GCCTCCCCCT AGGGTGGGCG

6121 AAGAACTCCA GCATGAGATC CCCGCGCTGG AGGATCATCC AGCCGGCGTC CCGGAAAACG

6181 ATTCCGAAGC CCAACCTTTC ATAGAAGGCG GCGGTGGAAT CGAAATCTCG TGATGGCAGG

6241 TTGGGCGTCG CTTGGTCGGT CATTTCGAAC CCCAGAGTCC CGCTCAGAAG AACTCGTCAA

6301 GAAGGCGATA GAAGGCGATG CGCTGCGAAT CGGGAGCGGC GATACCGTAA AGCACGAGGA

6361 AGCGGTCAGC CCATTCGCCG CCAAGCTCTT CAGCAATATC ACGGGTAGCC AACGCTATGT

6421 CCTGATAGCG GTCCGCCACA CCCAGCCGGC CACAGTCGAT GAATCCAGAA AAGCGGCCAT

6481 TTTCCACCAT GATATTCGGC AAGCAGGCAT CGCCATGGGT CACGACGAGA TCCTCGCCGT

6541 CGGGCATGCT CGCCTTGAGC CTGGCGAACA GTTCGGCTGG CGCGAGCCCC TGATGCTCTT

6601 GATCATCCTG ATCGACAAGA CCGGCTTCCA TCCGAGTACG TGCTCGCTCG ATGCGATGTT

6661 TCGCTTGGTG GTCGAATGG

//
